# Unscrambling variation in avian eggshell colour and patterning in a continent-wide study

**DOI:** 10.1098/rsos.181269

**Published:** 2019-01-30

**Authors:** Kiara L. L'Herpiniere, Louis G. O'Neill, Andrew F. Russell, Daisy Englert Duursma, Simon C. Griffith

**Affiliations:** 1 Department of Biological Sciences, Macquarie University, Sydney, New South Wales 2109, Australia; 2 Centre for Ecology and Conservation, University of Exeter Cornwall Campus, Penryn TR10 9FE, UK

**Keywords:** antimicrobial, brood parasite, maculation, signalling, solar radiation, structural

## Abstract

The evolutionary drivers underlying marked variation in the pigmentation of eggs within many avian species remains unclear. The leading hypotheses proposed to explain such variation advocate the roles of genetic differences, signalling and/or structural integrity. One means of testing among these hypotheses is to capitalize on museum collections of eggs obtained throughout a broad geographical range of a species to ensure sufficient variation in predictors pertaining to each hypothesis. Here, we measured coloration and patterning in eggs from 272 clutches of Australian magpies (*Cracticus tibicen*) collected across most of their geographical range of *ca* 7 million km^2^; encompassing eight subspecies, variation in environmental parameters, and the presence/absence of a brood parasite. We found considerable variation in background colour, as well as in the extent and distribution of patterning across eggs. There was little evidence that this variation was explained by subspecies or the contemporary presence of a brood parasite. However, measures of maximum temperature, leaf area index and soil calcium all contributed to variation in egg appearance, although their explanatory power was relatively low. Our results suggest that multiple factors combine to influence egg appearance in this species, and that even in species with highly variable eggs, coloration is not readily explained.

## 1. Introduction

Avian eggs vary widely across species in the expression of colour and patterning (hereafter termed maculation), arising from the deposition of varying concentrations of two pigments: protoporphyrin (brown) and biliverdin (blue/green) [1]. Traditionally, the ancestral avian egg was thought to be white [2]; very recently, however, the same two pigments have been found in non-avian dinosaur ancestors [3], suggesting that avian eggs evolved to be colourful millions of years ago. Variation in colour and maculation, be it in birds or dinosaurs, are likely to have evolved in response to one or more selective pressures [2,4]. Hypotheses that advocate such selective pressures broadly fall into signalling or structural functions [2,5], with the additional observation that there are often fixed, and presumably genetic differences across species and subspecies [6]. The suggested signalling functions include the use of colour to camouflage eggs from predators [7,8], to signal female quality [9–11] and as a marker enabling parents to distinguish their eggs from those laid by brood parasites [12]. By contrast, suggested structural functions of pigmentation include protection against microbes, and/or against extremes in temperature and solar radiation, as well as strengthening the eggshell [13–15]. There is significant support for each of these hypotheses (summarized in table 1) [2,5,13–33], but these mostly originate from interspecific comparative work [34–36], or local-scale studies within a single geographical location or population (e.g. [37–39]).

**Table 1.** Summary of the proposed hypotheses, key parameters, predictions, references related to each hypothesis, and examples of broad (B) or local (L) scales of studies.

hypothesis	rationale	prediction	references	broad (B)/local (L)
genetic differences	genetic isolation is expected to lead to variation due to random drift or local adaptation	eggs from each subspecies will cluster together with similar colours and/or patterning	[2,28]	(L) [28]
brood parasite hypothesis	variation in pigment use is driven by selection for host recognition of brood-parasitic eggs	eggs in the range of the brood parasite will show an absolute difference egg pigmentation and/or a change in variance	[2,5,29–32]	(B) [31,32] (L) [29]
bacterial hypothesis	eggshell pigments have antimicrobial properties; UV radiation triggers pigments to act as a natural defence against bacterial infection	eggs in warm and humid environments will be more pigmented	[13,16–18]	(L) [16,17]
solar radiation hypothesis	pigments aid in protecting embryos from overheating and solar irradiation; UV transmittance is lowest in brown eggs	eggs in areas of high solar radiation and low shade (i.e. arid zone) will be browner	[13,19–25]	(L) [20–22,24,25]
calcium availability hypothesis	avian eggshell comprises calcium carbonate; protoporphyrin (brown pigment in background and maculation) more prominent in calcium-poor environments; calcium availability to females can be related to calcium in the soil	eggs in calcium-poor areas (i.e. arid zone) will be more maculated	[14,15,26,27]	(L) [27,33]

While such approaches provide insight, they also have drawbacks. For example, the interspecific comparative approach typically condenses within-species variation into a single, species-specific value for analyses, which will not provide any insight into those species that are highly variable. With respect to the local-scale approach, many of the studies in the current literature have taken a largely univariate approach, testing just one, or a few, of the range of suggested hypotheses at a time, presumably partly because of the limited geographical scale available (table 1). Such studies are unable to deal with the potentially confounding influences caused by the inter-relationships between the range of biotic and abiotic parameters, and are therefore at risk of type two error, or a failure to correctly attribute the main determinant of variation. An intra-species but broad-scale approach, using clutches sampled over an extensive geographical range, provides enough variation in a variety of parameters to permit the simultaneous testing of each hypothesis. We here provide the first comprehensive examination of how egg colour and maculation vary, as well as their underlying predictors, over a continent-wide geographical range in a single species. We have taken both a univariate, and multivariate approach to specifically illustrate the limitations of the univariate approach in this field, even when examining quite clearly outlined hypotheses.

**Figure 1.**
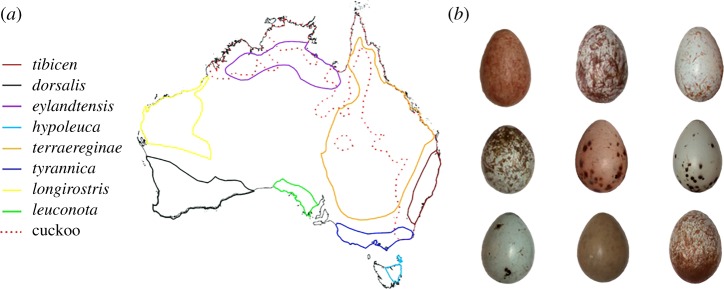
(*a*) Australian magpie (*Cracticus tibicen*) subspecies and channel-billed cuckoo (*Scythrops novaehollandiae*) distribution. Map digitized from the *Directory of Australian birds* [30]. (*b*) Example of variation in magpie eggs. Photographs were taken by KLL. All samples were from preserved museum collections.

We capitalize on museum collections of eggs of the Australian magpie (*Cracticus tibicen*) that had been collected throughout their *ca* 7 million km^2^ range encompassing most of the Australian continent (figure 1). This range captures eight subspecies, with about half of those overlapping with their only brood parasite, the channel-billed cuckoo (*Scythrops novaehollandiae*). While rates of parasitism, rather than the presence/absence of the parasite would be more informative, these data are unfortunately not available. Nevertheless, if the brood parasite does not exist in the range of a given subspecies, it cannot be currently parasitized, and so cannot contribute to the current selection pressure on the egg appearance of that subspecies. We recognize, however, that historic range expansions and contractions of this parasite, or indeed the presence of an additional brood parasite at some point in the past, will make these issues difficult to test.

The range we have sampled encompasses variation in temperature, humidity, vegetation and soil compositions at a continent-wide scale. We expect temperature and humidity to have direct effects on the survival of embryos, as well as indirect effects on microbial activity [13,16]. The habitat throughout this range varies from tropical and temperate rainforest through woodland, grassland and desert, and is expected to be captured by the leaf area index—defined as the average area of leaf cover between the ground and the sky in a vertical transect from the ground up [40]. Given that magpies are an arboreal nesting species, nests in areas with a lower leaf area index (open habitats) are more likely to be exposed to sunlight (less shade). The variation in habitat, therefore, can provide potential protection from harmful solar radiation exposure [1,13]. Finally, soil calcium levels, which are known to influence the structural integrity of eggs [22,41], vary considerably throughout the geographical range over which clutches were sampled. Thus, our sample encompasses variation in the key predictors of the main hypotheses proposed to explain variation in eggshell characteristics.

We have three specific aims. First, we quantify the variation in colour and maculation of eggs collected across the range of the Australian magpie, and the correlations among the traits measured. Second, we use a series of univariate analyses to investigate support for each hypothesis in turn; with these analyses taking the form of many of the other studies in the literature (e.g. [11,42], also for discussion see [23]). Specifically, we test whether variation in background colour and maculation can be explained: (i) at the subspecies level (i.e. by fixed genetic differences as a result of phenotypic divergence) [2]; (ii) by the presence/absence of the species' brood parasite—the channel-billed cuckoo (i.e. the signalling hypothesis through brood parasitism) [43]; and/or (iii) by temperature, relative humidity, leaf area index, soil calcium, all of which would be expected if egg variation was explained by selection on structural integrity [31]. Finally, we conduct a comprehensive multivariate analysis with all of the above biotic and abiotic parameters entered into a single model to investigate the relative support for hypotheses based on genetic differences, signalling and structural integrity (see table 1 for specific predictions). The comparison of the univariate approach and the more comprehensive multivariate approach will provide insight into the limitations of the simpler approach, when addressing a trait that is hypothesized to be affected by a range of different selection pressures simultaneously [2].

## 2. Material and methods

### 2.1. Egg collections

We accessed historic egg specimens of Australian magpies at the Victoria Museum in Melbourne and the Australian National Wildlife Collection (ANWC) in Canberra. Both collections were housed in the dry vertebrates’ collections in dark storage cabinets. The collection dates of the egg specimens ranged from 1862 to 1999. We selected clutches with known collection locations (latitude and longitude) and dates, giving a total sample size of 272 clutches. Museum accession numbers for each clutch, as well as the year of collection, geographical and taxonomic data are reported in electronic supplementary material, table S1.

### 2.2. Egg colour and maculation measures

Clutches were photographed in a standardized 40 × 40 cm studio photography light cube tent, on an egg holding surface. This surface was mounted with a colour reference card to process white balance and colour reflectance in the photographs, using a Canon 7D with a Sigma 18–250 mm lens in Victoria and a Canon E 7D with Canon Macro EfS 60 mm ultrasonic focal lens in Canberra (RAW format in both locations). All pictures were taken using a tripod and remote control for stability.

To measure the colour of eggs, we used standard spectrometry methods, which encompass the human visual and avian sensitivity in the UV part of the spectrum (300–700 nm). We used the UVS system for our analysis; the Australian magpie is part of the *Artamidae*, a diverse family centred in the Australian bioregion which also includes the *Paradisaeidea* (birds of paradise). While the magpie name would suggest it is in the *Corvidae* family, it is not. This is worth noting as some corvids are known only to have vision that is VS, rather than UVS sensitive [36]. Although we are unaware of studies of the optical sensitivity of any species within this largely Australasian family, it seems prudent to consider both a standard and UV model of vision in our analyses.

The use of spectrometry is both objective and repeatable [7]. We used a USB2000 + Miniature Fiber Optic spectrophotometer (Ocean Optics Inc., Dunedin, FL, USA), a xenon light source PX-2 (Ocean Optics Inc.) with a fibre-optic cable held at a 90° angle to the shells' surface, and reflectance data were recorded using the AvaSoft 7 program (Avantes, Eerbeek, The Netherlands). Colour measurements were taken from the pointed end of the egg, the median line of the egg (central part), and the base of the egg avoiding heavily maculated areas. Spectral measurements were taken by a different single measurer in each location—Victoria (LON), ANWC (SCG); no consistent differences were found between these two collections in any analysis (*p* = 0.31).

Variation in background colour was assessed using the *pavo* package in R [45] which allows the organization, visualization and analysis of spectral readings [46], as used in many colour analysis studies (e.g. [47–49]). Using this package, the readings were aggregated into a single value per egg. The processing functions within the package allowed the aggregated spectra to be loess-smoothed by a factor of 0.05 (see [46] for further details). A model was created based on different quantum catches at each photoreceptor for avian vision [50] (using avg.uv for ‘average avian UV system’, see *pavo* package for further detail on visual options) using the D65 ‘standard daylight’ background illuminant as Australian magpies are open cup nesters [6]. We chose the average avian UV system to approximate Australian magpie vision; however, we acknowledge that visual abilities differ between species [51] and the models embedded in *pavo* are intended only to approximate host perception.

The quantum catch outputs from this model were assessed through principal component analysis (PCA), built into the *robComposition* package [52]. The first principal component (PC1), which explained 69% of the variance in the quantum catch outputs, was used as our measure of egg colour. PC1 was negatively related to the variation in wavelength, with higher PC1 values being associated with blue reflectance curves, and lower values corresponding to brown reflectance curves (figure 3*a*). Biological pigments, especially biliverdin (responsible for the blue-green colour) have been shown to fade from the time they are laid to when they are later sampled in museum collections [53]. Recent research (e.g. [11]) has shown variation attributable to collection date to be negligible. We do, however, acknowledge that collection dates may have an effect on pigment fading, and we therefore included the year of collection in our models (as in [54]) to determine whether the age of eggs had any influence on colour, and found no significant effect.

To provide an objective, quantitative estimate of maculation, photographs were processed using an automated image-processing tool SpotEgg [55]. A number of digital image processors have been developed to quantify egg maculation [56], many of which focus primarily on the extent of parasitic egg mimicry [12,57,58]. We chose SpotEgg (run in Matlab v. 2012b) to measure maculation, as this program allows one to define the characteristics of the maculation (i.e. spots, blotches, clear) and so increases the precision with which the extent of maculation can be quantified in highly variable eggs (see electronic supplementary material, table S2 for configuration details). The total area of maculation, as a percentage of each egg's surface area was the response variable retained for our analyses.

### 2.3. Predictor variables

The Australian magpie is made up of eight subspecies and we sampled clutches from all subspecies. Based on the documentation provided with each clutch, these were: *Cracticus tibicen tibicen* (*N* = 54 clutches); *C. tibicen dorsalis* (*N* = 33); *C. tibicen eylandtensis* (*N* = 14); *C. tibicen hypoleuca* (*N* = 27); *C. tibicen terraereginae* (*N* = 58); *C. tibicen tyrannical* (*N* = 58); *C. tibicen longirostris* (*N* = 10) and *C. tibicen leuconota* (*N* = 18). A distribution map was extracted from the *Directory of Australian birds* [59] and digitized using ArcGIS. The distribution between magpie subspecies has considerable overlap, therefore a conservative contour map was created for each subspecies (figure 1). Of these eight subspecies, four are known to overlap the contemporary range of the channel-billed cuckoo [60] and were assigned to the ‘present’ category, while those that did not overlap were assigned to the ‘absent’ category, shown in figure 1.

**Figure 2.**
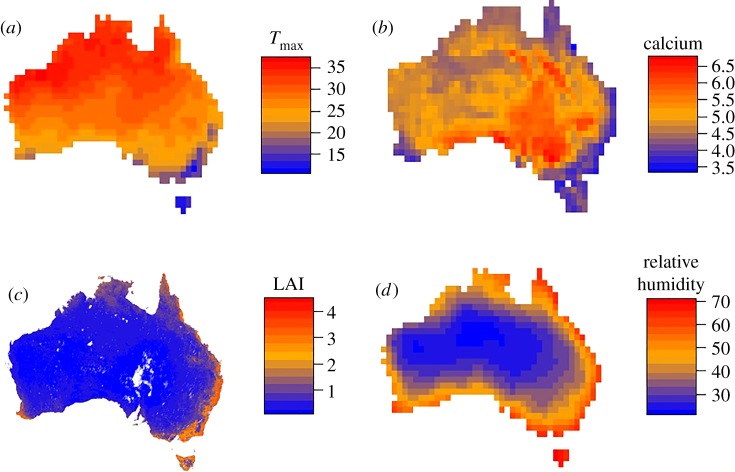
Maps showing variation in: (*a*) maximum temperatures (*T*_max_) based on a 30-year daily average; (*b*) calcium levels extracted from pH soil tests 0–5 cm deep; (*c*) leaf area index (LAI) based on average 16-year 16-day intervals and (*d*) relative humidity (%) based on a 30-year daily average for the Australian continent. Maps have been resampled to a 1° × 1° (100 × 100 km) grid cell resolution.

To investigate the relationship between ecological variables hypothesized to influence egg coloration and maculation, we calculated average daily maximum temperatures (*T*_max_, °C), relative humidity (%), leaf area index and soil calcium content for the Australian continent, at a 100 × 100 km grid cell resolution (figure 2). *T*_max_ and relative humidity were downloaded from the Australian Water Availability Project [61] via http://www.bom.gov.au. *T*_max_ was extracted as the maximum daily average for each grid cell from 1911 to 1940, while relative humidity was extracted as the maximum daily average at 15.00 for each grid cell from 1976 to 2005. These time periods are the earliest data available for the respective parameters [61] and the closest available to the time period when the eggs were collected. Leaf area index measurements represented the average for 16-day intervals in each cell during the period February 2000–2016 obtained via the TERN AusCover portal (http://www.auscover.org.au/) and produced from tiles originally downloaded from USGS (https://lpdaac.usgs.gov/). Soil calcium levels were extracted from the Soil and Landscape Grid of Australia via http://www.clw.csiro.au/aclep/soilandlandscapegrid in the form of 0–5 cm deep pH tests (CaCl_2_), with values averaged for each grid cell.

### 2.4. Statistical analyses

All analyses were carried out in R v. 3.3.2 [45]. In addition to R's ‘base functions’, we used the packages *maptools* [62], *raster* [63] and *visreg* [64] for data extraction, manipulation and visualization. Statistical tests were considered significant at an alpha-level of 0.05. We first tested whether variation in egg background colour and total area of maculation vary systematically among subspecies using an equality of variance (Levenes Test, package *car*) and an analysis of variance (ANOVA) test [65]. A Tukey's pair-wise comparison, in the package *multcomp* [66], was then conducted to elucidate which subspecies differed and which were comparable. We then conducted a series of eight mixed-effect model analyses (four each for background colour and maculation) to test the independent support for each hypothesis in turn (table 1). To test each hypothesis, the predictors (found in table 1) were fitted as explanatory terms, the collection year was included as a continuous variable to determine whether the age of the egg had any influence on colour, and unique grid number was fitted as a random term to account for repeated measures of clutches within the same grid cell. In each case, analyses were conducted using the package *lme4* [67], with subsequent use of *lmerTest* [68] and *MuMIn* [69] to generate *p*-values and *R*^2^-values, respectively. Moran's *I* statistic in the *spdep* package [70,71] was used to detect spatial autocorrelation of residuals. Where spatial autocorrelation was detected, a simultaneous autoregressive model (SAR) was used to add an autocorrected error term of spatial weights [70–72]*.* Finally, we conducted a multivariate analysis in which all explanatory terms outlined above were included in the same model. In this case, we used model reduction to generate a final model based on the anova function in R, with terms having non-significant explanatory power on the model being removed. Our aim here was to elucidate the relative support for each hypothesis simultaneously.

## 3. Results

### 3.1. Variation among subspecies

**Figure 3.**
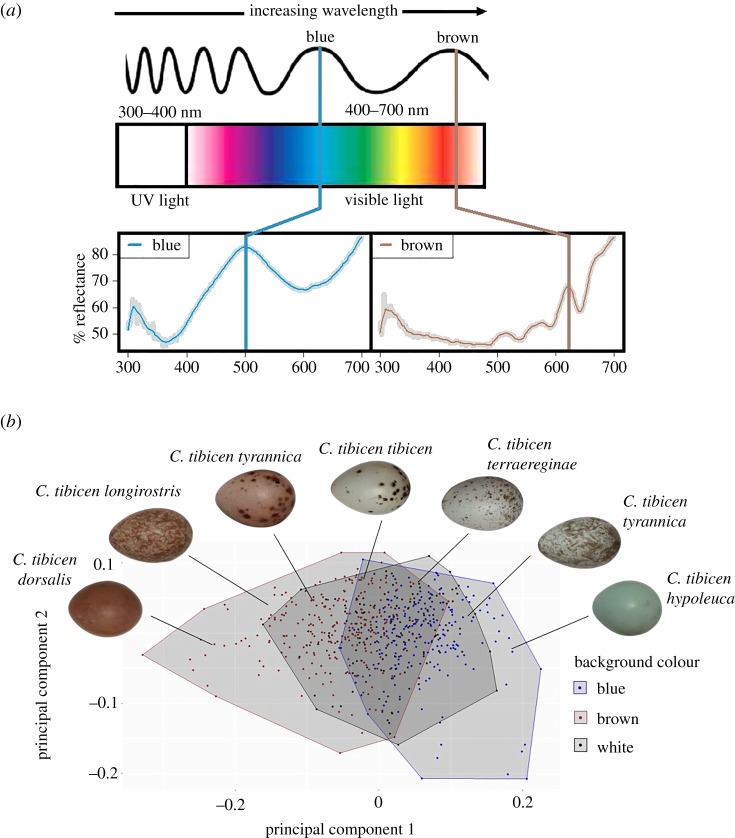
(*a*) Examples of reflectance curves returned from the spectrophotometer, between the wavelengths of 300 and 700 nm. On the left, the blue colour with the main peak at 500 nm and on the right the brown colour with the main peak at 630 nm. Both curves have been smoothed to remove noise using the loess-smooth function = 0.05. (*b*) Example of Australian magpie egg variety and their location in colour space when analysed with PCA. Data come from 272 clutches of eggs. Background colours (blue, brown and white) were visually marked in the museum and plotted to visualize where colours fell within the matrix.

We found extensive differences in the background colour of magpie clutches, with clutches varying from white to a variety of tones of blue, grey, brown and red (figure 3*a,b*). This variation was captured by two prominent reflectance peaks in the visible spectrum at 500 and 630 nm, reflecting blue and brown background colours, respectively (figure 3*a*,*b*). The eight subspecies showed comparable variation in egg coloration, indicated by a Levene's test of variance on PC1 (*F*_7,237_ = 0.73, *p* = 0.6). In addition, although we found significant average differences in background colour (PC1) among subspecies (one-way ANOVA *F*_7,245_ = 4.9, *p* < 0.001, *R*^2^ = 0.15), subsequent Tukey's tests suggested that this difference was driven entirely by *C. tibicen hypoleuca* (table 2). This Tasmanian subspecies had significantly bluer eggs than four of the seven other subspecies, and there were no systematic differences in the background colour among the other subspecies. Thus, population-wide consistent subspecies level genetic differences do not obviously explain the marked variation in background colour of Australian magpie eggs in the overall sample.

**Table 2.** Tukey multiple comparisons of means between principal component 1 (PC1) background colour values and subspecies of Australian magpies (*C. tibicen*). Eight subspecies and 245 clutches were analysed from preserved museum samples. All outliers from the specified distribution ranges were removed from the analysis (hence the reduced number of clutches in this specific analysis). Names in bold are those that differed most frequently. *p*-values show 95% family-wise confidence level. Italicized rows indicate a significant *p*-value. Direction of change indicates that *C. tibicen hypoleuca* has a higher PC1 value, thus bluer background colour, than four out of the seven other subspecies.

subspecies			estimate	s.e.	*z*	*p*
***hypoleuca***	*—*	*dorsalis*	*0.083*	*0.02*	*4.06*	*<0.005*
*longirostris*	*—*	***hypoleuca***	−*0.12*	*0.03*	−*3.73*	*<0.005*
*tyrannica*	*—*	*dorsalis*	*0.06*	*0.02*	*3.54*	*0.01*
*tyrannica*	*—*	*longirostris*	*0.1*	*0.03*	*3.29*	*0.02*
*leuconota*	*—*	***hypoleuca***	−*0.1*	*0.03*	−*3.21*	*0.03*
*tibicen*	*—*	***hypoleuca***	−0.055	0.019	−2.94	0.057
*terraereginae*	*—*	*Longirostris*	0.09	0.03	2.76	0.09

**Figure 4.**
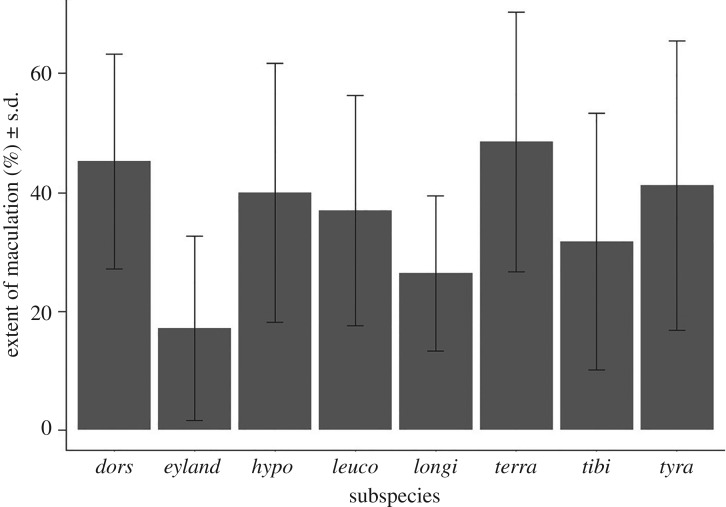
Barchart highlighting the extent of maculation of eggs of each subspecies (as a % of total surface area) with standard error. *Cracticus tibicen dorsalis* (mean = 45 ± 18%); *C. tibicen eylandtensis* (mean = 17 ± 16%); *C. tibicen hypoleuca* (mean = 40 ± 22%); *C. tibicen leuconota* (mean = 37 ± 19.5%); *C. tibicen longirostris* (mean = 26 ±13%); *C. tibicen terraereginae* (mean = 49 ± 22%); *C. tibicen* (mean = 32 ± 22%); *C. tibicen tyrannica* (mean = 41 ± 24.5%).

Maculation scores also varied in both extent and type of marking. Some eggs had almost no markings, and others were so heavily maculated it was challenging to see the background colour (figures 1 and 3*b*). Overall, the average area of maculation on Australian magpie eggs was 37% of the total surface (ranging from 0 to 85%, s.d. ± 21.5%). The eight subspecies showed potential variation in egg maculation, indicated by a Levene's test of variance on PC1, but any differences were slight (*F*_7,237_ = 1.9, *p* = 0.06). We did find more convincing evidence for significant mean differences in maculation scores among subspecies (one-way ANOVA *F*_7,272_ = 3.1, *p* < 0.001, *R*^2^ = 0.13), but again subsequent Tukey tests suggested this to be driven entirely by the maculation of a single subspecies, in this case *C. tibicen eylandtensis*. Eggs of this subspecies of the Northern Territory were significantly more maculated than three of the seven other subspecies with no systematic differences between the other subspecies (figure 4 and table 3). Again therefore, marked variation in maculation appeared not to be primarily driven by population-wide genetic differences that differ consistently across subspecies.

**Table 3.** Tukey multiple comparisons of means between the total area of maculation values (from SpotEgg) and subspecies of Australian magpies (*C. Tibicen*). Eight subspecies and 245 clutches were analysed from preserved museum samples. All outliers from the specified distribution ranges were removed from the analysis (table 2). Names in bold are those that differed most frequently. *p*-values show 95% family-wise confidence level. Italicized rows indicate a significant *p*-value. Direction of change indicates that *C. tibicen eylandtensis* has a lower total maculation value, thus spottier eggs than three of the seven other subspecies.

subspecies			estimate	s.e.	*z*	*p*
*terraereginae*	*—*	***eylandtensis***	*28.42*	*6.91*	*4.11*	*<0.001*
*tyrannica*	*—*	***eylandtensis***	*24.73*	*6.91*	*3.58*	*0.007*
***eylandtensis***	*—*	*dorsalis*	*−23.22*	*7.35*	*−3.16*	*0.03*
*tibicen*	*—*	*terraereginae*	*−*11.9	4.27	*−*2.79	0.08
*hypoleuca*	*—*	***eylandtensis***	20.68	7.46	2.77	0.09

### 3.2. Independent univariate tests of ecology-based hypotheses

We found little support for any of the hypotheses tested in the independent analyses of background colour (table 4). Eggs did not differ in colour between those subspecies living in the presence versus absence of the brood-parasitic channel-billed cuckoo. In addition, background colour did not vary as a function of the interaction between temperature and humidity, which might be expected given that microbial activity is likely to be greater in hot and humid climates. Further, the average level of soil calcium within each grid was not significantly associated with background colour, suggesting that colour variation does not serve to compensate for reduced calcium levels. Finally, we found no evidence of an effect of the interaction between temperature and leaf area index on background colour, as predicted by the solar radiation hypothesis.

**Table 4.** Results of hypothesis-based models investigating if principal component 1 (PC1) background colour values of Australian magpie eggs (*C. tibicen*) can be explained by: (*a*) the parasite hypothesis; (*b*) the bacterial hypothesis; (*c*) the solar radiation hypothesis or (*d*) the calcium hypothesis. Two hundred and seventy two clutches were analysed from museum samples. Where spatial autocorrelation was detected, it was corrected using SAR. The random variable in remaining models is the unique grid number (random UGN). The *R*^2^*m* reports the *R*^2^ of the model with just fixed effects while the *R*^2^*c* reports the *R*^2^ of the full model including random variables.

(*a*) parasite hypothesis					
*Background PC1 ∼ Presence/Absence; R*^2^*m* = 0.005, *R*^2^*c* =0.18					
	**estimate**	**s.e.**	**d.f.**	***t***	***p***
intercept	−5.87 × 10^−3^	2.16 × 10^−2^	1.7 × 10^+2^	−0.3	0.79
parasite yes	1.15 × 10^−2^	1.12 × 10^−2^	1.17 × 10^+2^	1.03	0.31
age	−6.36 × 10^−5^	2.21 × 10^−4^	1.54 × 10^+2^	−0.29	0.77
	**variance**	**s.d.**			
random UGN	0.001	0.03			
residuals	0.004	0.07			

(*b*) bacterial hypothesis					
*Background PC1 ∼ T_max_ ∼ r. humidity; R*^2^*m* = 0.05, *R*^2^*c* = 0.18					
	**estimate**	**s.e.**	**d.f.**	***t***	***p***
intercept	−3.3 × 10^−2^	1.3 × 10^−1^	5.95 × 10^+1^	−0.26	0.8
*T*_max_ : humid	4.84 × 10^−5^	9.98 × 10^−5^	7.24 × 10^+1^	−0.49	0.63
age	−8.7 × 10^−5^	2.13 × 10^−4^	1.36 × 10^+2^	−0.41	0.68
	**variance**	**s.d.**			
random UGN	0.0007	0.027			
residuals	0.005	0.07			

(*c*) solar radiation					
*Background PC1 ∼ T_max_ ∼ LAI; R*^2^*m* = 0.06, *R*^2^*c* = 0.2					
	**estimate**	**s.e.**	**d.f.**	***t***	***p***
intercept	1.24 × 10^−1^	6.41 × 10^−2^	5.36 × 10^+1^	1.93	0.06
LAI : *T*_max_	5.05 × 10^−4^	1.94 × 10^−3^	6.3 × 10^+1^	0.26	0.8
age	−2.01 × 10^−5^	2.15 × 10^−4^	1.43 × 10^+2^	−0.1	0.9
	**variance**	**s.d.**			
random UGN	0.001	0.03			
residuals	0.004	0.07			

(*d*) calcium					
*SAR* *Background PC1 ∼ calcium*					
	**estimate**	**s.e.**	***p***		
intercept	7.92 × 10^−1^	2.33 × 10^−1^	0.001		
calcium	4.54 × 10^−3^	6.69 × 10^−3^	0.5		
age	−4.32 × 10^−5^	2.07 × 10^−4^	0.84		

**Figure 5.**
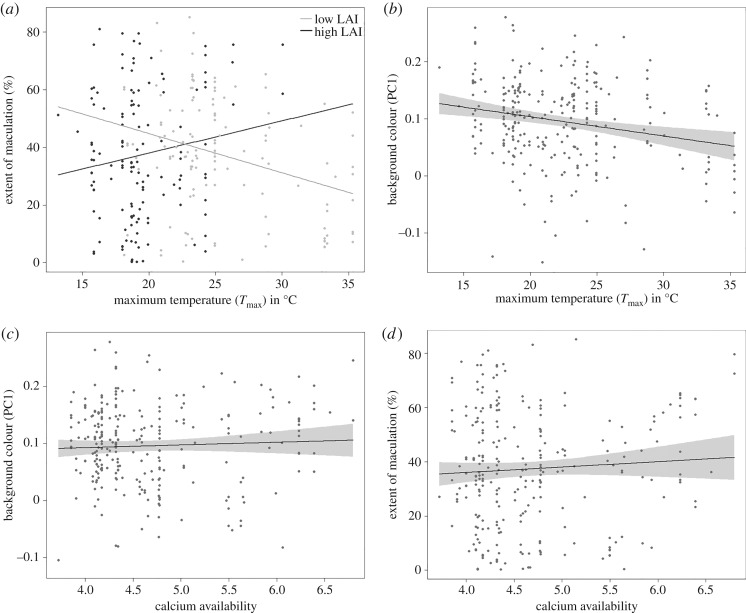
Interaction plots of ecological factors against background colour PC1 (high PC1 = blue, low PC1 = brown) or extent of maculation (%). Each point represents the average maculation % score or the average PC1 score of a clutch and the lines represent the best fits. (*a*) Interaction plot between maculation, maximum temperatures (*T*_max_) and LAI. Direction of change shows that eggs are more maculated in warmer and more shaded areas, and less so in cooler less shaded areas. (*b*) Relationship between PC1 and maximum temperature (*T*_max_). The direction of change indicates a lower PC1 value with higher temperature. (*c*) Relationship between PC1 and calcium availability. The direction of change indicates a higher PC1 value with higher calcium availability. (*d*) Relationship between maculation and calcium availability. Direction of change shows that eggs are more maculated in areas of higher calcium availability.

Similarly, we found little support for any of the hypotheses tested in the independent analyses of maculation (table 5). Again, levels of maculation were not associated with the presence or absence of channel-billed cuckoos. Nor were they associated with hot and humid climates or soil calcium levels, suggesting that maculation does not offer protection against microbial activity or compensate for low levels of soil calcium. Finally, although we found a significant relationship between the interaction between temperature and leaf area index and maculation (*F*_1.272_ = 6.8, *p* < 0.05), the direction of this interaction ran counter to the prediction of the solar radiation hypothesis (figure 5*a*). In other words, instead of eggs being more maculated in areas of high sun exposure, we found that they were more maculated in areas of high temperature and high (not low) leaf area index.

**Table 5.** Results of hypothesis-based models investigating if the total area of maculation (generated by SpotEgg) of Australian magpie eggs (*C. tibicen*) can be explained by (*a*) the parasite hypothesis; (*b*) the bacterial hypothesis; (*c*) the solar radiation hypothesis and (*d*) the calcium hypothesis. Two hundred and seventy two clutches were analysed from museum samples. The random variable included in all models is the unique grid number (random UGN). The *R*^2^*m* reports the *R*^2^ of the model with just fixed effects while the *R*^2^*c* reports the *R*^2^ of the full model including random variables. Italicized rows indicate a significant *p*-value.

(*a*) parasite hypothesis					
*Maculation scores ∼ Presence/Absence; R*^2^*m* = 0.004, *R*^2^*c* = 0.14					
	**estimate**	**s.e.**	**d.f.**	***t***	***p***
intercept	*32.5*	*6.12*	*175.7*	*5.3*	*<0.0001*
parasite yes	−0.17	3.14	117.9	−0.06	0.96
age	0.06	0.06	158.1	0.93	0.35
	**variance**	**s.d.**			
random UGN	61.54	7.8			
residuals	393.7	19.8			

(*b*) bacterial hypothesis					
*Maculation scores ∼ T*_max_ *∼ r. humidity; R*^2^*m* = 0.02, *R*^2^*c* = 0.11					
	**estimate**	**s.e.**	**d.f.**	***t***	***p***
intercept	*80.57*	*36.0*	*53.7*	*2.2*	*0.03*
*T*_max_ : r. humid	0.05	0.028	65.17	1.76	0.08
age	0.05	0.06	133.57	0.87	0.38
	**variance**	**s.d.**			
random UGN	39.9	6.32			
residuals	407.6	20.2			

(*c*) solar radiation					
*Maculation scores ∼ T_max_ ∼ LAI; R*^2^*m* = 0.04, *R*^2^*c* = 0.10					
	**estimate**	**s.e.**	**d.f.**	***t***	***p***
intercept	*74.0*	*16.9*	*39.8*	*4.4*	*<0.0001*
LAI : T_max_	*1.38*	*0.52*	*46.9*	*2.66*	*0.01*
age	0.06	0.06	131.4	1.06	0.3
	**variance**	**s.d.**			
random UGN	29.3	5.4			
residuals	410.84	20.27			

(*d*) calcium					
*Maculation scores ∼ calcium; R*^2^*m* = = 0.01, *R*^2^*c* = 0.14					
	**estimate**	**s.e.**	**d.f.**	***t***	***p***
intercept	18.9	12.3	127	1.54	0.12
calcium	2.61	2.07	126.4	1.26	0.21
age	0.07	0.06	157.2	1.14	0.25
	**variance**	**s.d.**			
random UGN	60.9	7.8			
residuals	391.5	19.79			

### 3.3. Multivariate analysis

Our multivariate approach was more successful at explaining significant variation in background colour. We found a positive relationship between background colour and *T*_max_, indicating that eggs are browner in locations with higher average maximum temperatures (*F*_1,272_ = 15.98, *p* < 0.001; table 6 and figure 5*b*). A significant relationship was also found between background colour and calcium availability in the soil (*F*_1,272_ = 6.44, *p* < 0.01), with eggs in areas of increased soil calcium being bluer (table 6 and figure 5*c*). Finally, we also detected a non-significant trend for the eggs of subspecies overlapping with channel-billed cuckoos to be bluer. By contrast, we found no evidence to suggest that background colour is influenced by relative humidity or leaf area index.

**Table 6.** Results of multivariate analysis whereby we investigate the explanatory power of environmental variables on background colour using principal component 1 (PC1). The final model includes *T*_max_, calcium and unique grid number (random UGN) as a random variable. The *R*^2^*m* reports the *R*^2^ of the model with just fixed effects while the *R*^2^*c* reports the *R*^2^ of the full model including random variables. Results suggest that as temperature increases PC1 decreases (gets browner) and as calcium increases PC1 increases (gets bluer). Italicized rows indicate a significant *p*-value. *R*^2^*m* = 0.082, *R*^2^*c* = 0.22.

	estimate	s.e.	d.f.	*t*	*p*
intercept	0.0097	0.036	82.52	0.27	
*T*_max_	*−0.0051*	*0.001*	*47.75*	*−4.1*	*0.0001*
calcium	*0.021*	*0.008*	*92.54*	*2.6*	*0.01*
parasite yes	0.02	0.011	82.00	1.69	0.096
relative humidity	0.001	0.001	85.4	1.25	0.22
LAI	0.016	0.014	47.03	1.15	0.26

	variance	s.d.			
random UGN	0.001	0.03			
residuals	0.005	0.07			

We also found significant ecological predictors of maculation scores with our multivariate analyses (table 7). As was reported in the more targeted analyses above, the interaction between maximum temperature and leaf area index was also significant in this analysis (*F*_1,272_ = 4.3, *p* < 0.05). In addition, however, we also detected a significant effect of calcium availability in the soil (*F*_1,272_ = 4.61, *p* < 0.05), although eggs were more, not less, maculated in areas of high calcium (figure 5*d*). Finally, we found no evidence for a role of cuckoo presence or relative humidity on egg maculation.

**Table 7.** Results of multivariate analysis whereby we investigate the explanatory power of environmental variables on maculation scores from SpotEgg. The final model includes *T*_max_, LAI, *T*_max_ * LAI (as an interaction) and unique grid number (random UGN) as a random variable. The *R*^2^*m* reports the *R*^2^ of the model with just fixed effects while the *R*^2^*c* reports the *R*^2^ of the full model including random variables. Results show that independently, as temperature increases, the total area of maculation decreases (gets less spotty) and as leaf area index increases (more leaf cover) total area of maculation decreases. However, when temperature and LAI increase together, the total area of maculation increases. Italicized rows indicate a significant *p*-value. *R*^2^*m* = 0.05, *R*^2^*c* = 0.13.

	estimate	s.e.	d.f.	*t*	*p*
intercept	34.33	25.29	64.32	1.36	0.18
*T*_max_	*−1.42*	*0.65*	*37.11*	*−2.19*	*0.03*
*T*_max_ * LAI	*6.43*	*2.99*	*79.02*	*2.15*	*0.03*
calcium	*1.08*	*0.51*	*56.93*	*2.07*	*0.04*
LAI	*−*18.84	12.63	67.23	*−*1.49	0.14
parasite yes	*−*2.99	3.15	80.54	*−*0.95	0.34
relative humidity	0.23	0.28	95.94	0.79	0.43

	variance	s.d.			
random UGN	39.61	5.88			
residuals	409.64	20.24			

## 4. Discussion

The eggs of Australian magpies are highly variable in background colour and in extent of maculation. Despite this, only the Tasmanian subspecies *C. tibicen hypoleuca*, differed in background colour, and only the Northern Territory subspecies *C. tibicen eylandtensis* differed in the extent of maculation. We found little compelling support for the hypotheses under investigation. Most notably, we did not find that eggs varied significantly in their appearance as a function of the current range of brood parasites or likely predictors of the microbial activity, calcium levels or solar radiation, at least as measured. We conclude that despite the number of environmental and biological parameters that we considered in our broad-scale analysis across the continent of Australia, most of the variation remains unexplained and multiple factors probably combine, perhaps to different extents in different areas, to influence egg colour and maculation in this species. In tentative support, our univariate analyses appeared less able to explain the observed variation than were our multivariate approaches. Despite this, many studies employ this single hypothesis based approach, which our study shows can lead to misleading results.

The Australian magpies' continent-wide distribution encompasses eight established subspecies showing differences in plumage, morphology and behaviour [6,18], perhaps suggesting prolonged periods of reproductive isolation. Such isolation might also expect to lead to divergence in egg characteristics arising from random genetic drift or local adaptation. Remarkably, we found that subspecies explained little of the marked variation in either egg coloration or extent of maculation across the species as a whole. Apart from the Tasmanian subspecies that produced eggs that were bluer on average than those of other subspecies, and the Northern Territory subspecies which laid more maculated eggs, all other subspecies showed comparable overlap in the extent of both background colour and the level of maculation. These results arose despite high levels of intra-clutch similarity in egg morphology (results not shown), perhaps suggesting high levels of heritability in eggshell colour traits. These results suggest either more fine-scaled selection on egg pigmentation within each subspecies (creating variance that breaks down any divergence between subspecies), or that any genetic polymorphism present within the species as a whole is not structured along the same lines as the subspecific variation.

Arguably, the most well-supported adaptive idea accounting for observed variation in egg morphology is the brood parasitism hypothesis [2,5,30–33]. However, we found only a weak trend for eggs to be bluer in areas where a parasite was present, which differs from the generally brown cuckoo egg. At best, this result suggests little selection on the part of the cuckoo to match its host eggs or little selection on the host to detect the cuckoo egg. In support of either, the Australian magpie is a secondary host of the channel-billed cuckoo, and the young cuckoo does not eject the host eggs on hatching [74]. As a consequence, we might expect reduced impacts of cuckoo parasitism on the egg morphology of Australian magpies (relative to other cuckoo species in which host egg or nestling rejection is absolute). Indeed, not only did background colour vary little between those subspecies living in the presence versus absence of cuckoos, but there were no differences in their levels of egg maculation. Thus, overall, we find little compelling support for the hypothesis that brood parasitism has shaped the marked variation in current egg colour variation in the Australian magpie.

Research on the anti-bacterial properties of egg pigments has shown that brown pigments have the most effective anti-bacterial properties when exposed to light (as most open nesting species’ eggs would be). For instance, Ishikawa *et al.* [16] found that photodynamic microbial properties of natural pigments reduced bacteria survival. Using domestic chicken eggs (*Gallus gallus domesticus*) and exposing them in standardized conditions to a treatment of bacteria, they found that brown eggs were more effective at bacterial defence when exposed to light than blue or white eggs [16]. Australia is characterized by substantial variation in climatic conditions. We hypothesized that microbial activity is greatest in hot and humid conditions—support for the antimicrobial function of egg pigmentation would thus be provided if eggs laid in such conditions are browner and/or more maculated (making use of more protoporphoryn). While we found that browner and more maculated eggs were more prevelant in areas of high temperature, we found no evidence that these temperature effects were modified by relative humidity levels. As a consequence, we found little firm evidence to suggest that the variation in egg pigmentation observed can be explained by selection against bacterial activity [16].

An alternative hypothesis that has been proposed to explain variation in maculation, but might also help to explain variation in background colour is the calcium deficiency hypothesis [22]. Calcium compounds are a key component of eggshells and are often limiting in the environment [41]. To compensate for any such limitations, females have been suggested to deposit increased levels of protoporphyrin primarily in the form of egg maculation. For example, Gosler *et al.* [15,22,27,41] found that in areas of low calcium, great tit (*Parus major*) eggs were more maculated. Australia is calcium poor compared with the environment in which this great tit study was conducted [22,75], leading to the expectation that magpie eggs should be highly maculated. Although many Australian magpie eggs were highly maculated, we found a positive, not negative, association between calcium levels in the environment and the extent of maculation. On the other hand, the background colour of eggs was more likely to be brown in areas with low calcium in the soil, which might confer greater structural integrity of eggs in calcium-poor environments. We fully acknowledge that omnivorous magpies might gain sufficient calcium from their diet, which might limit the utility of using measures of calcium in the environment to predict variation in egg coloration. It is therefore conceivable that variation in egg coloration reflects variation in the calcium levels found in the diet among different females. These potential issues notwithstanding, we also find little support for the calcium availability hypothesis in egg maculation, and the influence of background colour on the structural integrity of eggs is yet to be investigated by other studies, although our results suggest that it is worth testing.

That eggs were browner and more maculated when laid in areas of high average maximum temperatures provides some support for the solar radiation hypothesis. This hypothesis proposes that in areas of higher risk of harmful radiation, which is likely to correlate with the average maximum temperature, eggs should contain more protoporphyrin to protect them from this harmful UV radiation [13]. For example, experiments in chicken eggs show that embryonic exposure to UV is reduced when eggs have a higher intensity of brown pigmentation [13,76]. Given that magpies are uniparental incubators, eggs will be periodically exposed to direct sunlight during female recess bouts, leading to the potential for selection on increased protoporphyrin deposition in areas with higher exposure to sunlight. Although the need to increase the protoporphyrin content of the eggshells in areas of increased solar radiation might be expected to be modified by overall levels of foliage in the environment, this probably depends on the location of magpie nests with respect to foliage cover. Magpies typically position their nests high in a tree, usually only partly covered by any foliage and are exposed accordingly to the sun during female recess periods from incubation duty [73]. In addition, Australia has experienced significant aridification in the last 5 Myr and was more wooded when magpies evolved *ca* 7 Ma than it is today [77,78]. As a consequence, current measures of leaf area index might fail to capture variation in the amount of solar radiation experienced by ancestral magpie eggs, accounting for the lack of the expected interaction between temperature and leaf area index on background colour. Nevertheless, we found that eggs were more maculated when laid in areas with higher temperatures and leaf area index which runs counter to the hypothesis that increasing protoporphyrin content offers protection against solar radiation [5,21]. Together these results provide ambiguous support for the solar radiation hypothesis and more tests of the adaptive benefits of protoporphyrin use in background colour and patterning are required to understand the role of this selection pressure in egg colour evolution, particularly in areas with higher solar radiation.

In conclusion, although the eggs of the Australian magpie show marked variation in background colour and maculation, most of this variation remains unexplained, despite the number of environmental and biological parameters that we considered in our broad-scale analysis across the continent of Australia. In total, only 8% of the variation in background colour and 5% of the variation in maculation was explained by the range of explanatory variables that we considered. This is a disappointingly small amount of the variation that occurs in the highly variable eggs of this species, and our findings suggest that understanding the colour and patterning of birds eggs is not easy, as studies have previously suggested [2]. While selection driven by brood parasites has previously been found to explain significant amounts of the variation in some species [21], we found no support for this in the Australian magpie. A big caveat here is that our data and analyses were unable to account for historical selection pressures, driven by either past climatic or environmental conditions, or historical brood parasitism, either by the channel-billed cuckoo, or perhaps another unknown parasite. It therefore certainly remains possible that significant variation may have arisen as a result of past selection [68–70]. Finally, although the patterns were not very strong, and not always in the direction predicted, we did find some evidence of covariation between both background coloration and maculation with chemical and physical properties of the environment. The nature of these relationships suggests that both calcium and maximum temperature may play some role in contemporary selection on avian egg pigmentation, but that these patterns might be more complicated and less easy to predict than previous studies have indicated [41]. Our findings suggest that the selective role of calcium and maximum temperature are worthy of further investigation.

## Supplementary Material

Supplementary Materials for Magpie Egg Paper

## Data accessibility

Data available from the Dryad Digital Repository: http://dx.doi.org/10.5061/dryad.q3b7b78 [79].

## References

[1] Lahti DC, Ardia DR 2016 Shedding light on bird egg color: pigment as parasol and the dark car effect. Am. Nat. 187, 547–563. (10.1086/685780) 27104989

[2] Kilner RM 2006 The evolution of egg colour and patterning in birds. Biol. Rev. 81, 383–406. (10.1017/S1464793106007044) 16740199

[3] Wiemann J, Yang T-R, Norell MA 2018 Dinosaur egg colour had a single evolutionary origin. Nature 563, 555–558. (10.1038/s41586-018-0646-5) 30464264

[4] Reynolds SJ, Martin GR, Cassey P 2009 Is sexual selection blurring the functional significance of eggshell coloration hypotheses? Anim. Behav. 78, 209–215. (10.1016/j.anbehav.2009.03.003)

[5] Underwood T, Sealey S 2002 Adaptive significance of egg colouration. In Avian incubation: behaviour, environment, and evolution (ed. Deeming DC), pp. 280–297. Oxford, UK: Oxford University Press.

[6] Higgins P, Peter J, Cowling S 2006 Handbook of Australian, New Zealand & Antarctic birds, vol. 7 Melbourne, Australia: Oxford University Press.

[7] Stevens M 2011 Avian vision and egg colouration: concepts and measurements. Avian Biol. Res. 4, 168–184. (10.3184/175815511X13207790177958)

[8] Carnaby T 2008 Beat about the bush: birds. Johannesburg, South Africa: Jacana Media.

[9] Moreno J, Osorno JL 2003 Avian egg colour and sexual selection: does eggshell pigmentation reflect female condition and genetic quality? Ecol. Lett. 6, 803–806. (10.1046/j.1461-0248.2003.00505.x)

[10] Morales J, Sanz JJ, Moreno J 2006 Egg colour reflects the amount of yolk maternal antibodies and fledging success in a songbird. Biol. Lett. 2, 334–336. (10.1098/rsbl.2006.0471) 17148396 PMC1686209

[11] Hanley D, Cassey P, Doucet SM 2013 Parents, predators, parasites, and the evolution of eggshell colour in open nesting birds. Evol. Ecol. 27, 593–617. (10.1007/s10682-012-9619-6)

[12] Stoddard MC, Kilner RM, Town C 2014 Pattern recognition algorithm reveals how birds evolve individual egg pattern signatures. Nat. Commun. 5, 4117 (10.1038/ncomms5117) 24939367

[13] Maurer G, Portugal SJ, Cassey P 2011 Review: an embryo's eye view of avian eggshell pigmentation. J. Avian Biol. 42, 494–504. (10.1111/j.1600-048X.2011.05368.x)

[14] Solomon S 1997 Egg and eggshell quality. Ames, IA: Iowa State University Press.

[15] Gosler AG, Wilkin TA 2017 Eggshell speckling in a passerine bird reveals chronic long-term decline in soil calcium. Bird Study 64, 1–10. (10.1080/00063657.2017.1314448)

[16] Ishikawa S ichi, Suzuki K, Fukuda E, Arihara K, Yamamoto Y, Mukai T, Itoh M 2010 Photodynamic antimicrobial activity of avian eggshell pigments. FEBS Lett. 584, 770–774. (10.1016/j.febslet.2009.12.041) 20036664

[17] Walsberg GG, Schmidt CA 1992 Effects of variable humidity on embryonic development and hatching success of mourning doves. Auk 109, 309–314. (10.2307/4088199)

[18] Bruce J, Drysdale EM. 1994 Trans-shell transmission. In Microbiology of the avian egg (eds Board RG, Fuller R), pp. 63–91. London, UK: Chapman and Hall.

[19] Bakken GS, Vanderbilt VC, Buttemer WA, Dawson WR. 1978 Avian eggs: thermoregulatory value of very high near-infrared reflectance. Science 200, 321–323. (10.1126/science.200.4339.321) 17745564

[20] Bertram BCR, Burger AE. 1981 Are ostrich *Struthio camelus* eggs the wrong colour? Ibis 123, 207–210.

[21] Lahti DC 2008 Population differentiation and rapid evolution of egg color in accordance with solar radiation. Auk 125, 796–802. (10.1525/auk.2008.07033)

[22] Gosler AG, Higham JP, Reynolds SJ 2005 Why are birds’ eggs speckled? Ecol. Lett. 8, 1105–1113. (10.1111/j.1461-0248.2005.00816.x)

[23] Cassey P, Maurer G, Lovell PG, Hanley D 2011 Conspicuous eggs and colourful hypotheses: testing the role of multiple influences on avian eggshell appearance. Avian Biol. Res. 4, 185–195. (10.3184/175815511X13207699868421)

[24] Shafey TM, Ghannam MM, Al-Batshan HA, Al-Ayed MS. 2004 Effect of pigment intensity and region of eggshell on the spectral transmission of light that passes the eggshell of chickens. Int. J. Poult. Sci. 3, 228–233. (10.3923/ijps.2004.228.233)

[25] Gómez J, Ramo C, Stevens M, Liñán-Cembrano G, Rendón MA, Troscianko JT, Amat JA. 2018 Latitudinal variation in biophysical characteristics of avian eggshells to cope with differential effects of solar radiation. Ecol. Evol*.* 8, 8019–8029. (10.1002/ece3.4335) 30250681 PMC6144973

[26] Birkhead TR. 2016 The most perfect thing: inside (and outside) a bird's egg. London, UK: Bloomsbury.

[27] Gosler AG 2006 Yet even more ways to dress eggs. Br. Birds 99, 338–353.

[28] Wilkin TA, Gosler AG, Garant D, Reynolds SJ, Sheldon BC. 2009 Calcium effects on life-history traits in a wild population of the great tit (*Parus major*): analysis of long-term data at several spatial scales. Oecologia 159, 463–472. (10.1007/s00442-008-1222-8) 19034530

[29] Collias EC. 1993 Inheritance of egg-colour polymorphism in the village weaver (*Ploceus cucullatus*). Auk 110, 683–692.

[30] Cherry MI, Bennett ATD 2001 Egg colour matching in an African cuckoo, as revealed by ultraviolet-visible reflectance spectrophotometry. Proc. R. Soc. Lond. B 268, 565–571. (10.1098/rspb.2000.1414) PMC1088641 11297172

[31] Cherry MI, Gosler AG 2010 Avian eggshell coloration: new perspectives on adaptive explanations. Biol. J. Linn. Soc. 100, 753–762. (10.1111/j.1095-8312.2010.01457.x)

[32] Soler JJ, Møller AP 1996 A comparative analysis of the evolution of variation in appearance of eggs of European passerines in relation to brood parasitism. Behav. Ecol. 7, 89–94. (10.1093/beheco/7.1.89)

[33] Langmore NE et al 2005 The evolution of egg rejection by cuckoo hosts in Australia and Europe. Behav. Ecol. 16, 686–692. (10.1093/beheco/ari041)

[34] Stoddard MC, Yong EH, Akkaynak D, Sheard C, Tobias JA, Mahadevan L 2017 Avian egg shape: form, function and evolution. Science 356, 1249–1254. (10.1016/j.semcancer.2013.09.001.B-cell) 28642430

[35] Cassey P, Thomas GH, Portugal SJ, Maurer G, Hauber ME, Grim T, Lovell PG, Mikšík I 2012 Why are birds' eggs colourful? Eggshell pigments co-vary with life-history and nesting ecology among British breeding non-passerine birds. Biol. J. Linn. Soc. 106, 657–672. (10.1111/j.1095-8312.2012.01877.x)

[36] Cassey P, Portugal SJ, Maurer G, Ewen JG, Boulton RL, Hauber ME, Blackburn TM 2010 Variability in avian eggshell colour: a comparative study of museum eggshells. PLoS ONE 5, 1–9. (10.1371/journal.pone.0012054) PMC2918502 20711258

[37] Krist M, Grim T 2007 Are blue eggs a sexually selected signal of female collared flycatchers? A cross-fostering experiment. Behav. Ecol. Sociobiol. 61, 863–876. (10.1007/s00265-006-0315-9)

[38] De Coster G, De Neve L, Lens L. 2012 Intraclutch variation in avian eggshell pigmentation: the anaemia hypothesis. Oecologia 170, 297–304. (10.1007/s00442-012-2304-1) 22434407

[39] Hanley D, Heiber G, Dearborn DC 2008 Testing an assumption of the sexual-signaling hypothesis: does blue-green egg color reflect maternal antioxidant capacity? Condor 110, 767–771. (10.1525/cond.2008.8634)

[40] Watson D 1947 Comparative physiological studies on the growth of field crops: I. Variation in net assimilation rate and leaf area between species and varieties, and within and between years. Ann. Bot. 11, 41–76. (10.1093/oxfordjournals.aob.a083148)

[41] Gosler AG, Connor OR, Bonser RHC 2011 Protoporphyrin and eggshell strength: preliminary findings from a passerine bird. Avian Biol. Res. 4, 214–223. (10.3184/175815511X13207833399666)

[42] Duursma DE, Gallagher RV, Griffith SC 2017 Characterizing opportunistic breeding at a continental scale using all available sources of phenological data: an assessment of 337 species across the Australian continent. Auk 134, 509–519. (10.1642/AUK-16-243.1)

[43] Stoddard MC, Hauber ME 2017 Colour, vision and coevolution in avian brood parasitism. Phil. Trans. R. Soc. B 372, 20160339 (10.1098/rstb.2016.0339) 28533456 PMC5444060

[44] Ödeen A, Håstad O 2003 Complex distribution of avian color vision systems revealed by sequencing the SWS1 opsin from total DNA. Mol. Biol. Evol. 20, 855–861. (10.1093/molbev/msg108) 12716987

[45] R Core Team. 2017 R: A language and environment for statistical computing. Vienna, Austria: R Foundation for Statistical Computing.

[46] Maia R, Eliason CM, Bitton PP, Doucet SM, Shawkey MD 2013 pavo: An R package for the analysis, visualization and organization of spectral data. Methods Ecol. Evol. 4, 906–913. (10.1111/2041-210X.12069)

[47] Hanley D, Grim T, Cassey P, Hauber ME 2015 Not so colourful after all: eggshell pigments constrain avian eggshell colour space. Biol. Lett. 11, 20150087 (10.1098/rsbl.2015.0087) 25994009 PMC4455735

[48] Hanley D, Grim T, Igic B, Samaš P, Lopez AV, Shawkey MD, Hauber ME 2017 Egg discrimination along a gradient of natural variation in eggshell coloration. Proc. R. Soc. B 284, 20162592 (10.1098/rspb.2016.2592) PMC5310612 28179521

[49] Diniz P, Ribeiro PHL, Rech GS, Macedo RH 2016 Monochromatism, cryptic sexual dimorphism and lack of assortative mating in the rufous hornero, *Furnarius rufus albogularis*. Emu, 116, 294–300. (10.1071/MU15118)

[50] Endler JA, Mielke PW 2005 Comparing entire colour patterns as birds see them. Biol. J. Linn. Soc. 86, 405–431. (10.1111/j.1095-8312.2005.00540.x)

[51] Hart NS, Vorobyev M 2005 Modelling oil droplet absorption spectra and spectral sensitivities of bird cone photoreceptors. J. Comp. Physiol. A 191, 381–392. (10.1007/s00359-004-0595-3) 15711964

[52] Templ M, Hron K, Filzmoser P. 2011 robCompositions: an R-package for robust statistical analysis of compositional data. In Compositional data analysis: theory and application (eds V Pawlowsky-Glahn, A Buccianti), pp. 341–355. Chichester, UK: John Wiley & Sons Ltd.

[53] Cassey P, Maurer G, Duval C, Ewen JG, Hauber ME 2010 Impact of time since collection on avian eggshell colour: a comparison of museum and fresh egg specimens. Behav. Ecol. Sociobiol. 64, 1711 (10.1007/s00265-010-1027-8)

[54] Starling M, Heinsohn R, Cockburn A, Langmore N 2006 Cryptic gentes revealed in pallid cuckoos *Cuculus pallidus* using reflectance spectrophotometry. Proc. R. Soc. B 273, 1929–1934. (10.1098/rspb.2006.3490) PMC1634764 16822754

[55] Gómez J, Liñán-Cembrano G 2016 SpotEgg: an image-processing tool for automatised analysis of colouration and spottiness. J. Avian Biol. 48, 502–512. (10.1111/jav.01117)

[56] Ornés AS, Herbst A, Spillner A, Mewes W, Rauch M 2014 A standardized method for quantifying eggshell spot patterns. J. F. Ornithol. 85, 397–407. (10.1111/jofo.12079)

[57] Stoddard MC, Stevens M 2010 Pattern mimicry of host eggs by the common cuckoo, as seen through a bird's eye. Proc. R. Soc. B 277, 1387–1393. (10.1098/rspb.2009.2018) PMC2871939 20053650

[58] Medina I, Troscianko J, Stevens M, Langmore NE 2016 Brood parasitism is linked to egg pattern diversity within and among species of Australian passerines. Am. Nat. 187, 351–362. (10.1086/684627) 26913947

[59] Schodde R, Mason I 1999 Directory of Australian birds: passerines. Collingwood, Australia: CSIRO Publishing.

[60] Brooker MG, Brooker LC 1989 Cuckoo hosts in Australia. Aust. Zool. Rev. 2, 1–67.

[61] Jones DA, Wang W, Fawcett R 2009 High-quality spatial climate data-sets for Australia. Aust. Meteorol. Oceanogr. J. 58, 233–248. (10.22499/2.5804.003)

[62] Bivand R, Lewin-Koh N 2017 maptools: tools for reading and handling spatial objects. R package version 0.9-2.

[63] Hijmans RJ 2016 raster: geographic data analysis and modeling. R package version 2.5-8.

[64] Breheny P, Burchett W. 2016 visreg: visualization of regression models R package version 2.3-0.

[65] Fox J, Weisberg S 2011 An R companion to applied regression, 2nd edn. Thousand Oaks, CA: SAGE Publications, Inc.

[66] Hothorn T, Bretz F, Westfall P 2008 Simultaneous inference in general parametric models. Biometrical J. 50, 346–363. (10.1002/bimj.200810425) 18481363

[67] Bates D, Maechler M, Bolker B, Walker S 2015 Fitting linear mixed-effects models using lme4. J. Stat. Softw. 67, 1–48. (10.18637/jss.v067.i01.)

[68] Kuznetsova A, Brockhoff PB, Christensen RHB. 2017 lmerTest: tests in linear mixed effects models J. Stat. Softw. 82, 1–26. (10.18637/jss.v082.i13)

[69] Barton K 2016 MuMIn: multi-model inference.

[70] Bivand R, Hauke J, Kossowski T 2013 Computing the Jacobian in Gaussian spatial autoregressive models: an illustrated comparison of available methods. Geogr. Anal. 45, 150–179. (10.1111/gean.12008)

[71] Bivand R, Piras G 2015 comparing implementations of estimation methods for spatial econometrics. J. Stat. Softw. 63, 1–36.

[72] Dormann C et al 2007 Methods to account for spatial autocorrelation in the analysis of species distributional data: a review. Ecography (Cop.*).* 30, 609–628. (10.1111/j.2007.0906-7590.05171.x)

[73] Kaplan G 2004 Australian magpie: biology and behaviour of an unusual songbird. Collingwood, Australia: CSIRO Publishing.

[74] Davies NB 2000 Cuckoos, cowbirds and other cheats. London, UK: T & AD Poyser.

[75] Richter DDJ, Markewitz D 2001 Understanding soil change: soil sustainability over millennia, centuries, and decades. Camb. Univ. Press 19, 255 (10.1097/00010694-200210000-00008)

[76] Shafey TM, Al-Batshan HA, Ghannam MM, Al-Ayed MS 2005 Effect of intensity of eggshell pigment and illuminated incubation on hatchability of brown eggs. Br. Poult. Sci. 46, 190–198. (10.1080/00071660500065789) 15957439

[77] Jetz W, Thomas G, Joy J, Hartmann K, Mooers A 2012 The global diversity of birds in space and time. Nature 491, 444–448. (10.1038/nature11631) 23123857

[78] White ME 1994 After the greening, the browning of Australia. Kenthurst, Australia: Kangaroo Press.

[79] L'Herpiniere KL, O'Neill LG, Russell AF, Duursma DE, Griffith SC 2019 Data from: Unscrambling variation in avian eggshell colour and patterning in a continent-wide study *Dryad Digital Repository*. (10.5061/dryad.q3b7b78) PMC6366205 30800374

