# Correction to: ‘Unscrambling variation in avian eggshell colour and patterning in a continent-wide study’ (2019), by L'Herpiniere *et al.*

**DOI:** 10.1098/rsos.240670

**Published:** 2024-07-03

**Authors:** Kiara L. L'Herpiniere, Louis G. O'Neill, Andrew F. Russell, Daisy Englert Duursma, Simon C. Griffith

*R. Sco. open sci.* **6**, 181269 (Published online 30 January 2019). (https://doi.org/10.1098/rsos.181269)

Owing to a data manipulation error in the raw data, the values in our principal component analysis (PCA) were incorrectly assigned. The error was noted upon the continuation of the study, whereby the PCA axes ran in the opposite direction. While revisiting the data, a minor labelling (scribing) error was also detected, which misassigned a small number of clutches at the data manipulation stage, thus changing the sample size reflected in the revised abstract and methods section. While the error mainly affects the PC1 term, it has flow-on effects throughout the text (abstract, results, discussion, tables and figures). Consequently, we have made the necessary corrections by re-running the analysis and revising the relevant sections outlined below. We found evidence for differences in Australian Magpie egg background coloration in relation to the contemporary presence of a brood parasite (Channel-billed cukoo) when investigated alongside measures of maximum temperature, relative humidity, leaf area index and soil calcium.

## Summary of corrections

***Abstract:*** *Edit in sample size and findings.*

***§2.0. Methods:*** *Changes in PC1 variation % and direction of change (§2.2). Species-specific sample sizes (§2.3).*

***§3.0. Results:*** *Corrected F, p, R^2^ values and summary findings for the subspecies analysis (§3.1)—background colour (paragraph 1) and maculation (paragraph 2), univariate analysis (§3.2)—maculation (paragraph 2), multivariate analysis (§3.3)—background colour (paragraph 1) and maculation (paragraph 2).*

***§4.0. Discussion:*** *Corrected interpretations for overall study (paragraph 1), variation across subspecies (paragraph 2), brood parasitism hypothesis (paragraph 3), anti-microbial hypothesis (paragraph 4), calcium hypothesis (paragraph 5), solar radiation hypothesis (paragraph 6), general conclusion (paragraph 7).*

***Figures:*** *figure 3. (b) (eggs in colour space) – Figure replotted, and legend corrected. Figure 5. (results plots) have been replotted to reflect our findings, and figure legends have been updated.*

***Tables:*** *Results* tables 2–7 *have been corrected to reflect corrected results.*

***Supplementary Figures:*** *Figures have been added to illustrate results.*

***Data accessibility:*** *Clean data and code have been uploaded to existing repository.*

## Description of errors and changes

***Abstract:*** *Edit in sample size and findings.*

***§2.0. Methods:*** *Principal component axes were the main source of error in section §2.2., the published direction of change was stated as higher values of PC1 being associated with blue reflectance curves, and lower values corresponding to brown reflectance curves. The corrected version stated that higher PC1 values are associated with brown reflectance curves, and lower values correspond to blue reflectance curves. PC1 explanatory power changed from 69% to 67%. In section §2.3, minor changes in species-specific sample size, and final sample size was made due to errors in scribing (and subsequent mismatch between trait data and museum data): Cracticus tibicen tibicen (published N* *=* *54, corrected N* *=* *53 clutches); C. tibicen hypoleuca (published N* *=* *27, corrected N* *=* *28); C. tibicen terraereginae (published N* *=* *58, corrected N* *=* *56); C. tibicen tyrannica (published N* *=* *58, corrected N* *=* *60); C. tibicen longirostris (published N* *=* *10, corrected N* *=* *9). Change in overall sample size from 272 to 271.*

***§3.0. Results:*** *Analyses were re-run and values have been updated throughout the text and tables.*

*The subspecies analysis (§3.1): published background colour results indicated differences were driven entirely by a single species (C. tibicen hypoleuca). Corrected result indicates differences were driven by two species, C. tibicen hypoleuca* and *C. tibicen longirostris*. *In interpretation error was detected in the published maculation results: Published version states that eggs of C. tibicen eylandtensis were more maculated than four of the seven other subspecies when they are in fact less maculated (data correct, figure 4 shows lower maculation score- minor error not picked up at publication stage).*

*Univariate analysis (§3.2): Results for the univariate modelling remain unchanged, significant interaction for maculation F statistic updated.*

*Multivariate analysis (§3.3)—This section is where the bulk of the differences lie due to the directionality of the PC1 axes. The published results for background colour find a negative trend for T*_max_ *and positive trend for Calcium levels. The corrected results find a positive trend for T*_max_ *and negative trend for Calcium levels. Additionally, a negative trend was found in relation to parasite presence, and an interaction between humidity and LAI. Published maculation results indicate a significant relationship with T*_max_*, the interaction between T*_max_ *and LAI, and calcium levels. The first two findings remain the same (with values updated), however, the corrected analysis finds no trend for calcium levels and a relationship with humidity levels.*

***§4.0. Discussion:*** *There are minor differences in the interpretation for overall study.*

*Brood parasitism hypothesis: Published version states we found only a weak trend, corrected version states we found evident differences between parasitized and non-parasitized species.*

*Anti-microbial hypothesis: Published version states we found no evidence that temperature effect on background colour were modified by humidity levels. Corrected version states that we only found evidence of humidity effects in relation to LAI, and that maculation differed between humidity conditions. Published version states that we found little firm evidence that egg pigmentation can be explained by selection against bacterial activity, corrected version states that maculation may be providing an antimicrobial function.*

*Calcium hypothesis: published version states we found a relationship between maculation and calcium levels, corrected version finds no association.*

*Solar radiation hypothesis: published version states eggs were browner and more maculated, corrected version states eggs were browner only.*

***Figures:*** *figure 3. (b) (eggs in colour space)—This figure served as the indicator of an issue within the dataset as the direction of the PCA data were incorrect (high PC1 values were reported as being associated with blue eggs and low PC1 value with brown eggs). This has been replotted and legend corrected to show that high PC1 are associated with darker and low PC1 with lighter—which concurred with later publications.*

***Figure 5.** (Results plots) have been replotted to reflect our findings, and figure legends have been updated. The corrected version shows a change in direction of PC1 values (5b and 5c). The presence and absence of the parasite has been superimposed onto 5b. Panel 5d has been changed from maculation against calcium availability to maculation against temperature in relation to humidity.*

***Tables:*** *Results tables 2–7 have been corrected to reflect corrected results described above.*

## Abstract

—Here, we measured coloration and patterning in eggs from 271 clutches of Australian magpies (*Cracticus tibicen*).

—We found evidence for differences in background coloration in relation to the contemporary presence of a brood parasite when investigated alongside measures of maximum temperature, relative humidity, leaf area index and soil calcium.

## §2.0. Methods

§**2.1.** giving a total sample size of 271 clutches.

§**2.2.** The first principal component (PC1), which explained 67% of the variance in the quantum catch outputs, was used as our measure of egg colour. PC1 was negatively related to the variation in wavelength, with higher PC1 values being associated with brown reflectance curves, and lower values corresponding to blue reflectance curves.

**§2.3.** Cracticus tibicen tibicen (*N* = 53 clutches); *C. tibicen hypoleuca* (*N* = 28); *C. tibicen terraereginae* (*N* = 56); *C. tibicen tyrannica* (*N* = 60); *C. tibicen longirostris* (*N* = 9).

## §3.0. Results

**§3.1.** The eight subspecies showed comparable variation in egg coloration, indicated by Levene's test of variance on PC1 (*F*_7,236_ = 0.89, *p* = 0.51). In addition, although we found significant average differences in background colour (PC1) among subspecies (one-way ANOVA *F*_7,236_ = 6.15, *p* < 0.001, *R*^2^ = 0.15, electronic supplementary material, figure S1), subsequent Tukey's tests suggested that this difference was driven by *C. tibicen hypoleuca* and *C. tibicen longirostris* (table 2). The Tasmanian subspecies (*C. tibicen hypoleuca)* had significantly lower PC1 (lighter/bluer) eggs than four of the seven other subspecies, and the North-western subspecies (*C. tibicen longirostris)* had significantly higher PC1 (darker) eggs than four of the seven other subspecies.

**§3.1.** The eight subspecies showed variation in egg maculation, indicated by Levene's test of variance on PC1 (*F*_7,236_ = 2.3, *p* = 0.03). We found evidence for significant mean differences in maculation scores among subspecies (One-way ANOVA *F*_7,236_ = 4.1, *p* < 0.001, *R*^2^ = 0.10), but again subsequent Tukey's tests suggested this to be driven entirely by the maculation of a single subspecies, in this case, the Northern Territory subspecies *C. tibicen eylandtensis*. Eggs of this subspecies were significantly less maculated than four of the seven other subspecies with no systematic differences between the other subspecies (figure 4 and table 3).

**§3.2.** Finally, although we found moderate evidence that the interaction between temperature and leaf area index had a positive effect on maculation (*F*_1.262_ = 4.2, *p* < 0.05), the direction of this interaction ran counter to the prediction of the solar radiation hypothesis (figure 5*a*).

**§3.3.** Our final model included *T*_max_, soil calcium levels, the presence of a parasitic cuckoo, an interaction between humidity and LAI and unique grid number as a random variable (*F*_6,264_ = 6.7, *p* *<* 0.001, table 6). We found strong evidence that *T*_max_ had a positive effect on background colour, whereby eggs were darker/browner in locations with higher maximum temperatures (figure 5*b*). There was evidence that calcium availability was negatively associated with background colour, whereby eggs were lighter/bluer where calcium availability was higher (figure 5*c*). The data revealed that the eggs of subspecies overlapping with channel-billed cuckoos were lighter/bluer than their counterparts (figure 5*b*, electronic supplementary material, S2). Finally, there was a negative association between relative humidity, LAI and background colour (an interaction we had not factored into our predictions), whereby eggs were lighter/bluer in areas of high humidity and leaf area index (electronic supplementary material, figure S3).

**§3.3.** Our final model included *T*_max_, relative humidity, LAI, an interaction between *T*_max_ and humidity, and an interaction between *T*_max_ and LAI (*F*_5,265_ = 3.5, *p* *<* 0.005, table 7). Eggs were less maculated in location with high maximum temperatures and high leaf area indices (independently, electronic supplementary material, figure S4–S5). As was reported in the more targeted analyses above, the interaction between maximum temperature and leaf area index was positively related to maculation (electronic supplementary material, figure 5*a*). Eggs were more maculated in humid regions (electronic supplementary material, figure S6), however, the directionality of that relationship differed in hot and humid conditions (electronic supplementary material, figure 5*d*). Finally, we found no evidence for a role of cuckoo presence or calcium availability in the soil on egg maculation.

## §4.0. Discussion

**§4.0.** Despite this, only the Tasmanian subspecies *C. tibicen hypoleuca* and the North-western subspecies *C. tibicen longirostris*, differed in background colour, and only the Northern Territory subspecies *C. tibicen eylandtensis* differed in the extent of maculation. We found little compelling support for the hypotheses under investigation when testing independently. When using a multivariate approach, we found that eggs varied significantly in their appearance as a function of the current range of the brood parasitic cuckoo and in relation to predictors of the microbial activity, calcium levels and solar radiation, albeit not in accordance with our predictions.

**§4.0.** The Tasmanian subspecies produced eggs that were lighter/bluer than the other subspecies, the North-western subspecies produced darker eggs on average than those of other subspecies, and the northern territory subspecies laid markedly less maculated eggs. All other subspecies showed comparable overlap in the extent of both background colour and the level of maculation.

**§4.0.** Our data revealed an evident difference between eggs of species laid where the distribution of the brood parasitic Channel-billed cuckoo overlapped. Egg of these species were found to be lighter/ bluer than those outside of the cuckoo's range, which differs from the darker, brown cuckoo egg.

**§4.0.** The lack of host rejection may imply a reduced need for patterned signatures (for recognition) and explain why there were no noticeable differences in the levels of egg maculation. Thus, overall, we found some compelling support for the hypothesis that brood parasitism has shaped the marked variation in current egg colour variation in the Australian magpie.

**§4.0.** While we found that browner eggs were more prevalent in areas of high temperature, we found evidence that humidity effects were only significant in relation to LAI, whereby eggs were lighter/bluer in more humid areas, especially so with high LAI (more shaded). Maculation differed considerably between conditions—eggs were less maculated in high temperature and low humidity, and more maculated in high temperatures and high humidity conditions. As a consequence, we found little firm evidence to suggest that the variation in background colour can be explained by selection against bacterial activity [16], however increased maculation may be providing an antimicrobial function in hot and humid areas.

**§4.0.** we found no association between calcium levels in the environment and the extent of maculation.

**§4.0.** we found no support for the calcium availability hypothesis in egg maculation,

**§4.0.** That eggs were browner when laid in areas of high average maximum temperatures provides some support for the solar radiation hypothesis.

**§4.0.** In total, only 16% of the variation in background colour and 5% of the variation in maculation was explained by the range of explanatory variables that we considered.

**§4.0.** Selection driven by brood parasites has previously been found to explain significant amounts of the variation in some species [21], here, we provide additional support for this hypothesis in the Australian magpie.

**§4.0.** The nature of these relationships suggests that maximum temperature, calcium, relative humidity, leaf cover and the interactions between these variables may play some role in contemporary selection on avian egg pigmentation.

**§4.0.** Our findings suggest that the selective role of ecological variables is worthy of further investigation.

**Figure 3.**
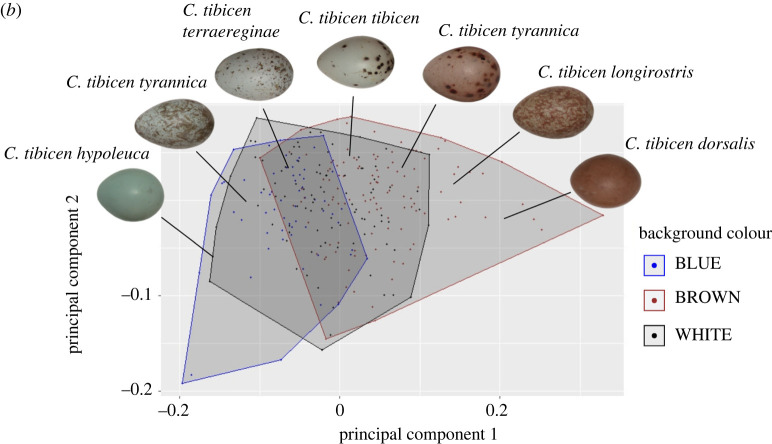
(*b*) Example of Australian magpie egg variety and their location in colour space when analysed with PCA. Data come from 271 clutches of eggs. Background colours (blue, brown and white) were visually marked in the museum and plotted to visualize where colours fell within the matrix.

**Figure 5.**
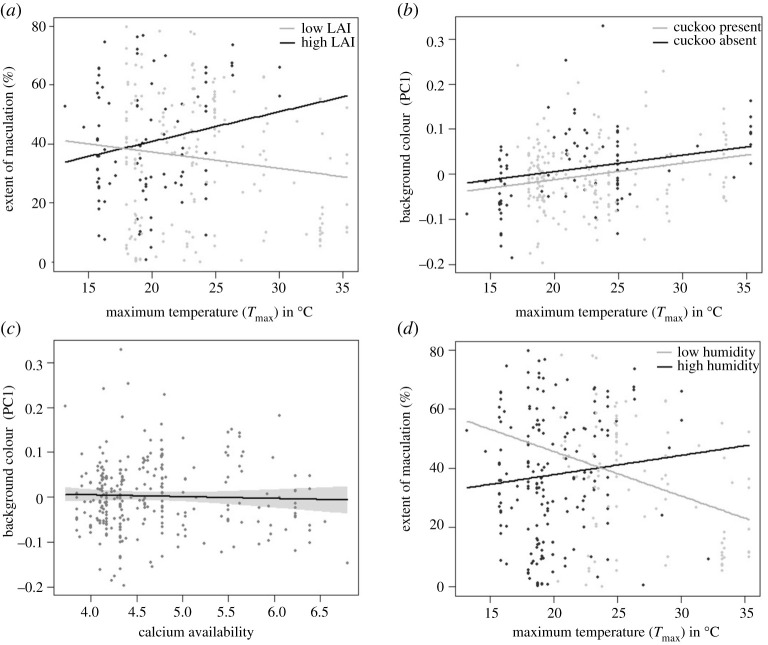
Interaction plots of ecological factors against background colour PC1 (high PC1 = brown, low PC1 = blue) or extent of maculation (%). Each point represents the average score of a clutch and the lines represent the best fits.

(a) Interaction between maculation, maximum temperatures (*T*_max_) and LAI. Direction of change shows that eggs are more maculated in warmer and more shaded areas, and less so in cooler less shaded areas.
(b) Relationship between PC1 and maximum temperature (*T*_max_) in the presence and absence of the brood-parasitic Channel-billed cuckoo. The direction of change indicates a higher PC1 value (darker eggs) with higher temperatures. Eggs laid within the cuckoos' distribution have lighter/bluer eggs (lower PC1) than those where the cuckoo is absent.
(c) Relationship between PC1 and calcium availability. The direction of change indicates a lower PC1 value with higher calcium availability.
(d) Interaction between maculation, maximum temperature (*T*_max_) and humidity. Direction of change shows that eggs are more maculated in hot and more humid areas, and less so in hot and dryer areas.

**Table 2.** Tukey multiple comparisons of means between principal component 1 (PC1) background colour values and subspecies of Australian magpies (*C. tibicen*). Eight subspecies and 244 clutches were analysed from preserved museum samples. All outliers from the specified distribution ranges were removed from the analysis (hence the reduced number of clutches in this specific analysis). Name in italics are those that differed most frequently. *p*-values show 95% family-wise confidence level, results presented are those with a *p*-value <0.05. Direction of change indicates that *C. tibicen hypoleuca* has a lower PC1 value (lighter, bluer background colour) than four out of the seven other subspecies, and *C. tibicen longirostris* has a higher PC1 value (darker background colour) than four out of the seven other subspecies.

subspecies			estimate	s.e.	*t*	*p*-values
*longirostris*	–	*Hypoleuca*	0.13	0.029	4.61	<0.001
*hypoleuca*	–	dorsalis	−0.08	0.019	−4.15	0.0011
tyrannica	–	*longirostris*	−0.11	0.028	−4.11	0.0013
tyrannica	–	dorsalis	−0.06	0.016	−3.52	0.011
leuconota	–	*hypoleuca*	0.10	0.029	3.49	0.012
terraereginae	–	*longirostris*	−0.10	0.028	−3.47	0.013
*hypoleuca*	–	eylandtensis	−0.08	0.024	−3.07	0.044
tibicen	–	*longirostris*	−0.08	0.028	−3.04	0.046

**Table 3.** Tukey multiple comparisons of means between the total area of maculation values (from SpotEgg) and subspecies of Australian magpies (*C. Tibicen*). Eight subspecies and 244 clutches were analysed from preserved museum samples. All outliers from the specified distribution ranges were removed from the analysis (see table 2 above). Names in italics are those that differed most frequently. *p*-values show 95% family-wise confidence level, italicized rows indicate a *p*-value <0.05. Direction of change indicates that *C. tibicen eylandtensis* has a lower total maculation value, thus less spotty eggs than four of the seven other subspecies.

subspecies			estimate	s.e.	*t*	*p*-values
terraereginae	–	*eylandtensis*	27.59	6.21	4.45	*<0.001*
*eylandtensis*	–	dorsalis	−24.96	6.56	−3.81	*0.0037*
tyrannica	–	*eylandtensis*	22.87	6.15	3.72	*0.0053*
hypoleuca	–	*eylandtensis*	22.32	6.69	3.34	*0.019*
tibicen	–	terraereginae	−11.76	3.99	−2.95	0.061

**Table 4.** Results of hypothesis-based models investigating if principal component 1 (PC1) background colour values of Australian magpie eggs (*C. tibicen*) can be explained by: (a) the parasite hypothesis; (b) the bacterial hypothesis; (c) the solar radiation hypothesis; or (d) the calcium hypothesis. In total, 271 clutches were analysed from museum samples. No evidence of spatial autocorrelation was found. A unique grid number (random UGN) was fitted to each model as a random variable. The $R_{m}^{2}$ reports the *R*^2^ of the model with just fixed effects while the $R_{c}^{2}$ reports the *R*^2^ of the full model including random variables.

(a) parasite hypothesis					
*background PC1* ∼ *presence/absence;* $R_{m}^{2}=0.005$, $R_{c}^{2}=0.23$					
	**estimate**	**s.e.**	**d.f.**	***t***	***p*-values**
intercept	2.19 × 10^−3^	2.14 × 10^−2^	184.47	0.10	0.92
parasite yes	−1.08 × 10^−2^	1.14 × 10^−2^	121.90	−0.95	0.34
age	8.71 × 10^−5^	2.18 × 10^−4^	175.36	0.40	0.69
	**variance**	**s.d.**			
random UGN	0.001	0.03			
residuals	0.004	0.07			
**(b) bacterial hypothesis**					
*background PC1* ∼ *T*_max_ * r. humidity; $R_{m}^{2}=0.05$, $R_{c}^{2}=0.22$					
	**estimate**	**s.e.**	**d.f.**	***t***	***p***
intercept	4.31 × 10^−2^	1.34 × 10^−1^	71.10	0.32	0.75
*T*_max_: humid	5.41 × 10^−5^	1.03 × 10^−4^	86.74	0.53	0.6
age	1.27 × 10^−4^	2.13 × 10^−4^	159.04	0.60	0.55
	**variance**	**s.d.**			
random UGN	0.001	0.031			
residuals	0.004	0.07			
**(c) solar radiation**					
*background PC1* ∼ *T*_max_ * LAI; $R_{m}^{2}=0.05$, $R_{c}^{2}=0.2$					
	**estimate**	**s.e.**	**d.f.**	***t***	***p-*values**
intercept	−1.05 × 10^−1^	6.20 × 10^−2^	64.46	−1.69	0.10
LAI : *T*_max_	−4.58 × 10^−5^	1.91 × 10^−3^	77.47	−0.024	0.98
age	7.66 × 10^−5^	2.14 × 10^−4^	163.05	0.36	0.72
	**variance**	**s.d.**			
random UGN	0.001	0.03			
residuals	0.004	0.07			
**(d) calcium**					
*background PC1* ∼ *calcium;* $R_{m}^{2}=0.01$, $R_{c}^{2}=0.25$					
	**estimate**	**s.e.**	**d.f.**	***t***	***p*-values**
intercept	4.34 × 10^−2^	4.46 × 10^−2^	148.60	0.97	0.33
calcium	−8.87 × 10^−3^	7.65 × 10^−3^	140.61	−1.16	0.25
age	1.42 × 10^−5^	2.20 × 10^−4^	177.55	0.064	0.95
	**variance**	**s.d.**			
random UGN	0.001	0.04			
residuals	0.004	0.06

**Table 5.** Results of hypothesis-based models investigating if the total area of maculation (generated by SpotEgg) of Australian magpie eggs (*C. tibicen*) can be explained by (a) the parasite hypothesis; (b) the bacterial hypothesis; (c) the solar radiation hypothesis; (d) the calcium hypothesis. In total, 271 clutches were analysed from museum samples. No evidence of spatial autocorrelation was found. A unique grid number (random UGN) was fitted to each model as a random variable. The $R_{m}^{2}$ reports the *R*^2^ of the model with just fixed effects while the $R_{c}^{2}$ reports the *R*^2^ of the full model including random variables. Italicized values indicate a *p*-value < 0.05.

(a) parasite hypothesis					
*maculation scores* ∼ *presence/absence;* $R_{m}^{2}=0.009$, $R_{c}^{2}=0.17$					
	**estimate**	**s.e.**	**d.f.**	***t***	***p-*value**
intercept	31.26	5.77	189.10	5.42	1.8 × 10^−7^
parasite yes	−1.55	3.03	121.93	−0.51	0.61
age	0.08	0.06	176.77	1.39	0.17
	**variance**	**s.d.**			
random UGN	66.59	8.16			
residuals	344.13	18.55			
**(b) bacterial hypothesis**					
*maculation scores* ∼ *T*_max_ * r. humidity; $R_{m}^{2}=0.02$, $R_{c}^{2}=0.15$					
	**estimate**	**s.e.**	**d.f.**	***t***	***p-*values**
intercept	66.99	35.92	66.72	1.87	0.07
*T*_max_ : r. humid	0.04	0.03	82.27	1.34	0.18
age	0.07	0.06	157.79	1.19	0.24
	**variance**	**s.d.**			
random UGN	54.35	7.37			
residuals	352.74	18.78			
**(c) solar radiation**					
*maculation scores* ∼ *T*_max_ * LAI; $R_{m}^{2}=0.03$, $R_{c}^{2}=0.15$					
	**estimate**	**s.e.**	**d.f.**	***t***	***p-*values**
intercept	61.51	15.93	53.56	3.86	0.0003
LAI : *T*_max_	1.03	0.50	66.33	2.07	*0.042*
age	0.07	0.06	157.92	1.31	0.19
	**variance**	**s.d.**			
random UGN	47.25	6.87			
residuals	355.04	18.84			
**(d) calcium**					
*maculation scores* ∼ *calcium;* $R_{m}^{2}=0.01$, $R_{c}^{2}=0.17$					
	**estimate**	**s.e.**	**d.f.**	***t***	***p-*values**
intercept	21.46	11.86	141.35	1.81	0.07
calcium	1.79	2.03	135.15	0.88	0.38
age	0.09	0.06	175.83	1.47	0.14
	**variance**	**s.d.**			
random UGN	67.6	8.2			
residuals	342.6	18.51

**Table 6.** Results of multivariate analysis investigating the explanatory power of environmental variables on background colour PC1. All variables were fitted to the full model, including a unique grid number (as a random variable, UGN) before performing automatic backwards elimination. The $R_{m}^{2}$ reports the *R^2^* of the model with just fixed effects while the $R_{c}^{2}$ reports the *R^2^* of the full model including random variables. The final model includes *T*_max_, the presence of a parasite, soil calcium levels, an interaction between relative humidity * LAI, and UGN as a random variable. Results suggest that PC1 increases (darker colour) with *T*_max_; PC1 decreases (lighter/bluer colour) in the presences of a parasite; PC1 decreases with increased calcium levels; and decreases with the interaction between relative humidity and LAI. Italicized values indicate a *p*-value < 0.05. $R_{m}^{2}=0.16$, $R_{c}^{2}=0.34$.

	estimate	s.e.	d.f.	*t*	*p**-*****values**
intercept	−4.11 × 10^−2^	7.46 × 10^−2^	89.58	−0.55	0.58
*T*_max_	7.38 × 10^−3^	1.55 × 10^−3^	67.01	4.75	*1.10 × 10^−5^*
parasite yes	−2.85 × 10^−2^	1.15 × 10^−2^	102.21	−2.49	*1.46 × 10^−2^*
calcium	−2.64 × 10^−2^	1.11 × 10^−2^	110.98	−2.39	*1.87 × 10^−2^*
r. humid * LAI	−2.88 × 10^−3^	1.40 × 10^−3^	61.93	−2.05	*4.42 × 10^−2^*
LAI	2.41 × 10^−2^	1.60 × 10^−2^	80.22	1.51	0.14
relative humidity	−3.08 × 10^−4^	1.68 × 10^−3^	87.61	−0.18	0.85
	**variance**	**s.d.**			
random UGN	0.001	0.03			
residuals	0.004	0.07

**Table 7.** Results of multivariate analysis investigating the explanatory power of environmental variables on maculation scores from SpotEgg. All variables were fitted to the full model, including a unique grid number (as a random variable, UGN) before performing automatic backwards elimination. The *R^2^a* reports the adjusted *R^2^* of the model, accounting for the number of predictors in the model. The final model includes *T*_max_, LAI, relative humidity, the interactions between *T*_max_ * LAI and *T*_max_ * relative humidity. Results suggest that as *T*_max_ and LAI increase (independently), the total area of maculation decreases (less spotty), as relative humidity increases (independently), the total area of maculation increases. However, the total area of maculation increases with the interaction between *T*_max_ and LAI, and decreases with the interaction between *T*_max_ and relative humidity. Italicized values indicate a *p*-value < 0.05. $R_{a}^{2}=0.05$.

	estimate	s.e.	*t*	*p-*values
intercept	71.89	13.93	5.16	4.85 × 10^−7^
*T*_max_	−1.45	0.52	−2.78	*0.006*
LAI	−33.13	10.36	−3.20	*0.002*
relative humidity	3.56	1.18	3.02	*0.003*
*T*_max_ * LAI	1.49	0.46	3.26	*0.001*
*T*_max_ * r.humid	−0.14	0.05	−2.97	*0.003*
	**d.f.**			
residual s.e.	20.13	265		

**Supplementary figure:**
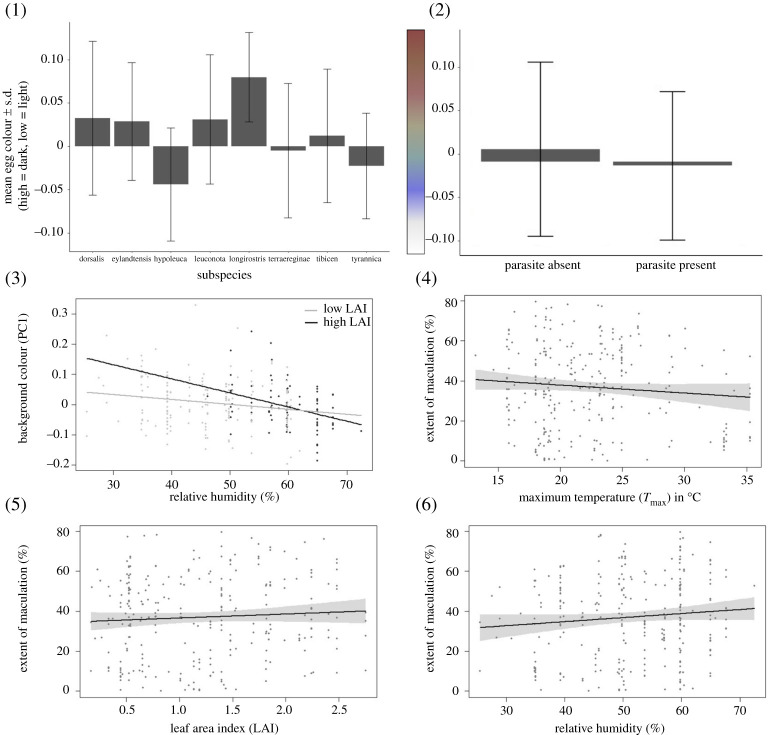
(1) Mean egg colour ± s.d. (high values are associated with darker/browner pigments and lower values are associated with lighter/bluer pigmentation) plotted by subspecies of Australian Magpie. Results indicate *C. tibicen hypoleuca* has lower PC1 and *C. tibicen longirostris* has a higher PC1; (2) mean egg colour ± s.d. plotted according to species overlap with parasitic Channel-billed Cuckoo. Species with overlap has a lower PC1 than those who do not; (3) egg background colour (PC1) in relation to relative humidity in conditions of high and low leaf area index (LAI). PC1 decreases with humidity, more so in more shaded conditions; (4) extent of maculation (as a % surface cover) in relation to maximum temperatures (*T*_max_). Maculation decreases with increased temperatures; (5) extent of maculation (%) in relation to LAI. Maculation increases with increased leaf cover; (6) extent of maculation (%) in relation to relative humidity (%). Maculation increases with relative humidity.

## Data accessibility

Data and code have been uploaded to the existing Dryad Digital Repository associated with original publication: https://doi.org/10.5061/dryad.q3b7b78.

## Declaration of AI use

We have not used AI-assisted technologies in creating this article.

