# Supplementary material for: Unscrambling variation in avian eggshell colour and patterning in a continent-wide study
Source: R Soc Open Sci. 2019 Jan 30;6(1):181269. doi: 10.1098/rsos.181269 (PMC6366205; doi:10.1098/rsos.181269)
Supplement: Supplementary Materials for Magpie Egg Paper [file rsos181269supp1.docx]

# SUPPLEMENTARY MATERIALS

| **SM TABLE 1:** Museum accession numbers (Reg. Number), year and location of original collection and Museum code (VIC = Victoria museum in Melbourne,  ANWC = Australian National Wildlife Collection in Canberra) | | | | | | | | | |
| --- | --- | --- | --- | --- | --- | --- | --- | --- | --- |
| Reg. Number | Year | Latitude | Longitude | Museum | Reg. Number | Year | Latitude | Longitude | Museum |
| 3542 | 1908 | -31.98 | 151.12 | VIC | E00830 | 1952 | -35 | 149 | ANWC |
| 3543 | 1894 | -29.7 | 152.93 | VIC | E00831 | 1952 | -35 | 149 | ANWC |
| 3544 | 1899 | -36.78 | 145.17 | VIC | E00832 | 1952 | -35 | 149 | ANWC |
| 3545 | 1893 | -29.7 | 152.93 | VIC | E00833 | 1952 | -35 | 149 | ANWC |
| 3546 | 1897 | -23.72 | 149.67 | VIC | E00847 | 1952 | -35 | 149 | ANWC |
| 3547 | 1906 | -31.98 | 151.12 | VIC | E00886 | 1952 | -35 | 149 | ANWC |
| 3548 | 1876 | -34.82 | 149.68 | VIC | E00887 | 1952 | -35 | 149 | ANWC |
| 3549 | 1909 | -31.82 | 151.32 | VIC | E00888 | 1952 | -35 | 149 | ANWC |
| 3550 | 1893 | -29.7 | 152.93 | VIC | E00889 | 1952 | -35 | 149 | ANWC |
| 3551 | 1897 | -23.72 | 149.67 | VIC | E00890 | 1952 | -35 | 149 | ANWC |
| 3552 | 1894 | -29.7 | 152.93 | VIC | E00892 | 1952 | -35 | 149 | ANWC |
| 3553 | 1897 | -23.72 | 149.67 | VIC | E02001 | 1912 | -32 | 149 | ANWC |
| 3554 | 1876 | -34.82 | 149.68 | VIC | E02002 | 1920 | -32 | 149 | ANWC |
| 3555 | 1892 | -29.7 | 152.93 | VIC | E02003 | 1920 | -32 | 149 | ANWC |
| 3556 | 1876 | -34.82 | 149.68 | VIC | E02004 | 1913 | -31 | 116 | ANWC |
| 3557 | 1907 | -31.98 | 151.12 | VIC | E02005 | 1913 | -32 | 149 | ANWC |
| 3558 | 1892 | -29.7 | 152.93 | VIC | E02029 | 1920 | -32 | 149 | ANWC |
| 3559 | 1893 | -29.7 | 152.93 | VIC | E02058 | 1911 | -31 | 116 | ANWC |
| 3560 | 1894 | -29.7 | 152.93 | VIC | E03606 | 1930 | -27 | 153 | ANWC |
| 3561 | 1907 | -31.82 | 151.32 | VIC | E04467 | 1948 | -34 | 146 | ANWC |
| 3562 | 1897 | -23.72 | 149.67 | VIC | E04470 | 1948 | -34 | 146 | ANWC |
| 3563 | 1908 | -19.33 | 146.47 | VIC | E05726 | 1956 | -35 | 139 | ANWC |
| 3564 | 1894 | -29.7 | 152.93 | VIC | E05728 | 1953 | -35 | 139 | ANWC |
| 3565 | 1909 | -31.82 | 151.32 | VIC | E05734 | 1947 | -35 | 139 | ANWC |
| 3566 | 1909 | -31.82 | 151.32 | VIC | E05735 | 1954 | -34 | 151 | ANWC |
| 3567 | 1909 | -31.82 | 151.32 | VIC | E05736 | 1955 | -34 | 118 | ANWC |
| 3568 | 1909 | -32.08 | 150.98 | VIC | E05737 | 1960 | -33 | 116 | ANWC |
| 3569 | 1909 | -31.82 | 151.32 | VIC | E05738 | 1961 | -33 | 116 | ANWC |
| 3570 | 1909 | -31.82 | 151.32 | VIC | E05739 | 1975 | -29 | 138 | ANWC |
| 3571 | 1909 | -31.98 | 151.12 | VIC | E05740 | 1982 | -35 | 139 | ANWC |
| 3572 | 1909 | -17.5 | 145.47 | VIC | E05741 | 1979 | -29 | 139 | ANWC |
| 3573 | 1910 | -31.87 | 141.42 | VIC | E05743 | 1981 | -29 | 139 | ANWC |
| 3574 | 1910 | -31.62 | 144.98 | VIC | E05747 | 1954 | -35 | 140 | ANWC |
| 3575 | 1911 | -17.5 | 145.47 | VIC | E05748 | 1954 | -35 | 140 | ANWC |
| 3576 | 1911 | -29.33 | 148.67 | VIC | E05750 | 1957 | -35 | 141 | ANWC |
| 3577 | 1899 | -33.38 | 149.48 | VIC | E05751 | 1957 | -35 | 141 | ANWC |
| 3578 | 1908 | -33.83 | 151.07 | VIC | E05752 | 1960 | -32 | 138 | ANWC |
| 3579 | 1899 | -33.38 | 149.48 | VIC | E05753 | 1944 | -38 | 140 | ANWC |
| 3580 | 1895 | -33.42 | 149.57 | VIC | E05756 | 1960 | -35 | 138 | ANWC |
| 3581 | 1899 | -33.38 | 149.48 | VIC | E05759 | 1956 | -33 | 138 | ANWC |
| 3582 | 1913 | -16.07 | 136.3 | VIC | E05760 | 1953 | -33 | 138 | ANWC |
| 3583 | 1913 | -16.07 | 136.3 | VIC | E05762 | 1971 | -35 | 138 | ANWC |
| 3584 | 1913 | -16.07 | 136.3 | VIC | E05764 | 1956 | -35 | 138 | ANWC |
| 3585 | 1913 | -16.07 | 136.3 | VIC | E06294 | 1999 | -33 | 146 | ANWC |
| 3586 | 1913 | -16.07 | 136.3 | VIC | E06337 | 1999 | -35 | 149 | ANWC |
| 3587 | 1913 | -18.6 | 136.1 | VIC | E06426 | 1918 | -37 | 150 | ANWC |
| 3588 | 1913 | -18.6 | 136.1 | VIC | E06427 | 1918 | -37 | 150 | ANWC |
| 3589 | 1913 | -18.6 | 136.1 | VIC | E06428 | 1918 | -37 | 150 | ANWC |
| 3590 | 1913 | -18.6 | 136.1 | VIC | E06429 | 1918 | -37 | 150 | ANWC |
| 3591 | 1913 | -18.6 | 136.1 | VIC | E06468 | 1960 | -37 | 144 | ANWC |
| 3592 | 1913 | -18.6 | 136.1 | VIC | E07851 | 1921 | -43 | 147 | ANWC |
| 3593 | 1890 | NA | NA | VIC | E07852 | 1933 | -43 | 147 | ANWC |
| 3594 | 1914 | -32.08 | 150.37 | VIC | E07853 | 1927 | -43 | 147 | ANWC |
| 3595 | NA | -32.07 | 149.23 | VIC | E07854 | 1928 | -43 | 148 | ANWC |
| 3596 | 1917 | -35.17 | 141.7 | VIC | E07855 | 1926 | -43 | 147 | ANWC |
| 3597 | 1921 | -13.95 | 143.2 | VIC | E07856 | 1925 | -43 | 147 | ANWC |
| 3598 | 1921 | -13.95 | 143.2 | VIC | E07857 | 1931 | -43 | 147 | ANWC |
| 3599 | 1906 | -17.32 | 123.63 | VIC | E07858 | 1921 | -43 | 147 | ANWC |
| 3600 | 1908 | -20.68 | 119.65 | VIC | E07859 | 1921 | -43 | 147 | ANWC |
| 3601 | 1908 | -20.68 | 119.65 | VIC | E07860 | 1911 | -41 | 147 | ANWC |
| 3602 | 1907 | -34.32 | 118.45 | VIC | E07861 | 1927 | -43 | 147 | ANWC |
| 3603 | 1908 | -20.68 | 119.65 | VIC | E07863 | 1924 | -43 | 147 | ANWC |
| 3604 | 1908 | -20.68 | 119.65 | VIC | E07864 | 1925 | -43 | 147 | ANWC |
| 3605 | 1908 | -20.68 | 119.65 | VIC | E07865 | 1950 | -42 | 147 | ANWC |
| 3606 | 1908 | -20.68 | 119.65 | VIC | E08398 | 1900 | -33 | 145 | ANWC |
| 3607 | 1907 | -34.32 | 118.45 | VIC | E08403 | 1905 | -33 | 149 | ANWC |
| 3608 | 1907 | -34.32 | 118.45 | VIC | E09982 | 1910 | -24 | 150 | ANWC |
| 3609 | 1903 | -31.95 | 115.85 | VIC | E10177 | 1908 | -35 | 141 | ANWC |
| 3610 | 1909 | -30.63 | 116 | VIC | E10178 | 1908 | -35 | 141 | ANWC |
| 3611 | 1909 | -30.63 | 116.02 | VIC | E10180 | 1915 | -31 | 116 | ANWC |
| 3612 | 1910 | -30.63 | 116.02 | VIC | E10182 | 1905 | -36 | 138 | ANWC |
| 3613 | 1912 | -30.63 | 116 | VIC | E10183 | 1906 | -38 | 144 | ANWC |
| 3614 | 1912 | -30.63 | 116 | VIC | E10184 | 1906 | -37 | 142 | ANWC |
| 3615 | 1912 | -30.63 | 116 | VIC | E10187 | 1906 | -35 | 142 | ANWC |
| 3616 | 1912 | -30.63 | 116 | VIC | E10188 | 1908 | -43 | 147 | ANWC |
| 3617 | 1912 | -30.63 | 116 | VIC | E13137 | 1959 | -28 | 152 | ANWC |
| 3618 | 1912 | -30.63 | 116 | VIC | E13138 | 1959 | -28 | 152 | ANWC |
| 3619 | 1912 | -30.63 | 116.02 | VIC | E13139 | 1962 | -20 | 147 | ANWC |
| 3620 | 1912 | -30.63 | 116 | VIC | E13140 | 1967 | -20 | 147 | ANWC |
| 3621 | 1904 | -37.83 | 144.57 | VIC | E13141 | 1984 | -26 | 145 | ANWC |
| 3622 | 1897 | -37.83 | 144.57 | VIC | E13142 | 1986 | -29 | 142 | ANWC |
| 3623 | 1896 | -37.62 | 144.35 | VIC | E13391 | 1949 | -34 | 150 | ANWC |
| 3624 | 1893 | -36.52 | 142.42 | VIC | E13463 | 1926 | -35 | 150 | ANWC |
| 3625 | 1904 | -37.83 | 144.57 | VIC | E13464 | 1926 | -35 | 150 | ANWC |
| 3626 | 1899 | -36.17 | 145.88 | VIC | E13465 | 1926 | -35 | 147 | ANWC |
| 3627 | 1913 | -34.92 | 141.03 | VIC | E14139 | 1961 | -35 | 149 | ANWC |
| 3628 | 1918 | -38.17 | 144.18 | VIC | E14140 | 1978 | -34 | 115 | ANWC |
| 3629 | 1917 | -38.17 | 144.18 | VIC | E14804 | 1975 | -30 | 117 | ANWC |
| 3630 | 1919 | -37.78 | 145.32 | VIC | E14805 | 1958 | -35 | 139 | ANWC |
| 3631 | 1919 | -37.03 | 141.28 | VIC | E14806 | 1963 | -35 | 139 | ANWC |
| 3632 | 1898 | -42.83 | 147.28 | VIC | E14811 | 1972 | -30 | 146 | ANWC |
| 3633 | 1907 | -42.43 | 145.62 | VIC | E14812 | 1932 | -34 | 151 | ANWC |
| 3634 | 1905 | -41.35 | 147.25 | VIC | E15488 | 1904 | -38 | 144 | ANWC |
| 3635 | 1914 | -42.75 | 147.23 | VIC | E15489 | 1924 | -38 | 144 | ANWC |
| 3636 | 1919 | -41.42 | 147.13 | VIC | E15490 | 1919 | -38 | 144 | ANWC |
| 3637 | 1914 | -42.53 | 147.2 | VIC | E15491 | 1919 | -38 | 144 | ANWC |
| 3638 | 1914 | -42.93 | 147.48 | VIC | E15492 | 1922 | -38 | 144 | ANWC |
| 3639 | 1914 | -42.75 | 147.23 | VIC | E15493 | 1907 | -38 | 145 | ANWC |
| 3640 | 1908 | -42.93 | 147.48 | VIC | E15494 | 1922 | -38 | 144 | ANWC |
| 5246 | NA | NA | NA | VIC | E15495 | 1905 | -36 | 145 | ANWC |
| 5247 | NA | NA | NA | VIC | E15496 | 1919 | -38 | 144 | ANWC |
| 5255 | NA | NA | NA | VIC | E15497 | 1931 | -38 | 144 | ANWC |
| 6752 | 1889 | -33.63 | 115.15 | VIC | E15498 | 1922 | -38 | 144 | ANWC |
| 6755 | 1885 | -23.9 | 149.5 | VIC | E15871 | 1937 | -22 | 144 | ANWC |
| 6756 | 1908 | -20.68 | 119.65 | VIC | E15901 | 1931 | -38 | 144 | ANWC |
| 6766 | NA | -41.55 | 147.28 | VIC | E18168 | 1904 | -33 | 149 | ANWC |
| 6774 | NA | NA | NA | VIC | E18182 | 1926 | -31 | 116 | ANWC |
| 6783 | NA | -38.08 | 144.27 | VIC | E18193 | 1905 | -36 | 149 | ANWC |
| 6791 | 1886 | -37.72 | 144.83 | VIC | E18231 | 1903 | -33 | 149 | ANWC |
| 6799 | NA | NA | NA | VIC | E18232 | 1909 | -41 | 147 | ANWC |
| 7624 | NA | NA | NA | VIC | E18233 | 1906 | -33 | 149 | ANWC |
| 7630 | NA | NA | NA | VIC | E18234 | 1905 | -36 | 149 | ANWC |
| 7636 | 1951 | -37.6 | 144.25 | VIC | 16227 | 1923 | -35.17 | 141.68 | VIC |
| 9051 | 1930 | -36.7 | 144.27 | VIC | 16229 | 1923 | -35.17 | 141.68 | VIC |
| 9052 | 1920 | -37.55 | 143.85 | VIC | 16230 | 1923 | -35.17 | 141.68 | VIC |
| 9053 | 1920 | -37.55 | 143.85 | VIC | 16231 | 1922 | -37.77 | 145.35 | VIC |
| 9054 | 1920 | -37.55 | 143.85 | VIC | 16232 | 1922 | -37.77 | 145.35 | VIC |
| 9055 | 1961 | -33.25 | 115.83 | VIC | 16233 | 1918 | -37.8 | 145.03 | VIC |
| 9056 | 1966 | -33.33 | 115.63 | VIC | 16451 | 1906 | -31.95 | 141.45 | VIC |
| 9057 | NA | NA | NA | VIC | 16462 | 1903 | -37.58 | 141.4 | VIC |
| 9059 | NA | -17.27 | 145.6 | VIC | 16597 | 1930 | -36.9 | 144.22 | VIC |
| 9060 | 1954 | -35.07 | 142.32 | VIC | 17140 | 1917 | -35.5 | 138.7 | VIC |
| 9260 | 1964 | -17.27 | 145.6 | VIC | 17141 | 1917 | -29.47 | 149.85 | VIC |
| 9261 | 1934 | -35.62 | 144.12 | VIC | 17142 | NA | -30.63 | 116 | VIC |
| 9263 | 1916 | -35.12 | 142.8 | VIC | 17143 | NA | -18.6 | 136.1 | VIC |
| 9264 | 1907 | -35.43 | 145.72 | VIC | 17144 | 1917 | -42.75 | 147.28 | VIC |
| 9265 | 1917 | -36.72 | 141.83 | VIC | 17524 | 1929 | -30.28 | 115.03 | VIC |
| 9266 | 1910 | -34.33 | 117.92 | VIC | 18227 | NA | -36.62 | 143.27 | VIC |
| 9267 | 1909 | -41.4 | 147.13 | VIC | 18228 | NA | -36.62 | 143.27 | VIC |
| 9268 | 1918 | -37.97 | 144.5 | VIC | 18412 | NA | NA | NA | VIC |
| 9270 | 1914 | -38.12 | 147.07 | VIC | 18522 | NA | -37.83 | 144.57 | VIC |
| 9271 | 1914 | -42.53 | 147.2 | VIC | 18613 | 1897 | NA | NA | VIC |
| 9272 | 1917 | -30.63 | 116 | VIC | 18645 | 1889 | NA | NA | VIC |
| 9273 | 1905 | -38.22 | 145.17 | VIC | 13754 | 1898 | NA | NA | VIC |
| 9274 | 1906 | -37.7 | 144.47 | VIC | 13757 | NA | -12.45 | 130.83 | VIC |
| 9275 | 1907 | -35.47 | 143.63 | VIC | 16225 | 1923 | -35.17 | 141.68 | VIC |
| 9276 | 1919 | -30.63 | 116 | VIC | 16226 | 1923 | -35.17 | 141.68 | VIC |
| 9277 | 1917 | -36.72 | 141.83 | VIC | 11801 | NA | NA | NA | VIC |
| 9278 | 1919 | -37.97 | 144.5 | VIC | 11808 | 1895 | -29.7 | 152.93 | VIC |
| 9279 | 1912 | -35.43 | 143.63 | VIC | 12338 | NA | NA | NA | VIC |
| 9280 | 1917 | -37.85 | 147.08 | VIC | 12342 | NA | NA | NA | VIC |
| 9281 | 1913 | -35.47 | 143.63 | VIC | 13080 | 1913 | -18.6 | 136.1 | VIC |
| 9282 | 1918 | -37.92 | 144.45 | VIC | 13081 | 1893 | -23.72 | 149.67 | VIC |
| 9283 | 1917 | -35.47 | 143.63 | VIC | 13740 | NA | -31.9 | 115.97 | VIC |
| 9284 | 1909 | -35.25 | 141.1 | VIC | 13741 | NA | NA | NA | VIC |
| 9285 | 1909 | -22.05 | 116.88 | VIC | 13742 | 1866 | -35.3 | 149.67 | VIC |
| 9286 | 1917 | -35.47 | 143.63 | VIC | 13743 | 1864 | -34 | 150.92 | VIC |
| 9287 | 1911 | -30.07 | 116.13 | VIC | 13744 | 1862 | -34 | 150.92 | VIC |
| 9288 | 1906 | -33.38 | 148.02 | VIC | 13747 | NA | NA | NA | VIC |
| 9289 | 1914 | -38.12 | 147.07 | VIC | 9292 | 1918 | -37.92 | 144.45 | VIC |
| 9290 | 1919 | -37.78 | 145.32 | VIC | 9293 | 1919 | -37.97 | 144.5 | VIC |
| 9291 | 1919 | -37.78 | 145.32 | VIC |  |  |  |  |  |

| **SM TABLE 2:** SpotEgg configurations settings for spot detection and linearization for three categories of eggs. Settings are determined by the training interface incorporated within SpotEgg. Reflection values refer to the colour reference card to process white balance and colour reflectance. | | | | |
| --- | --- | --- | --- | --- |
| Setting type | Radius Filter | Sensitivity | Minimum spot size | Background fill threshold |
| Spot | 1.01 | 0.080389 | 0.5 | 0.5 |
| Blotch | 0.62 | 0.035479 | 0.5 | 0.5 |
| Clear | 0.74 | 0.20872 | 0.5 | 0.5 |
| Reflectance values using DKG5x7 | 0.649000, 0.346000, 0.226800, 0.137800, 0.078900, 0.037000 | | | |
